# A membrane transporter determines the spectrum of activity of a potent platinum–acridine hybrid anticancer agent

**DOI:** 10.1038/s41598-020-72099-z

**Published:** 2020-09-16

**Authors:** Xiyuan Yao, Noah H. Watkins, Heather Brown-Harding, Ulrich Bierbach

**Affiliations:** 1 Department of Chemistry, Wake Forest University, Wake Forest Innovation Quarter, 455 Vine St., Winston-Salem, NC 27101 USA; 2 Department of Biology, Wake Forest University, Wake Forest Innovation Quarter, 455 Vine St., Winston-Salem, NC 27101 USA; 3 Comprehensive Cancer Center, Wake Forest School of Medicine, Medical Center Boulevard, Winston-Salem, NC 27157 USA

**Keywords:** Bioinorganic chemistry, Drug discovery and development, Screening, Target identification, Target validation

## Abstract

Cytotoxic drugs that are mechanistically distinct from current chemotherapies are attractive components of personalized combination regimens for combatting aggressive forms of cancer. To gain insight into the cellular mechanism of a potent platinum–acridine anticancer agent (compound **1**), a correlation analysis of NCI-60 compound screening results and gene expression profiles was performed. A plasma membrane transporter, the solute carrier (SLC) human multidrug and toxin extrusion protein 1 (hMATE1, *SLC47A1*), emerged as the dominant predictor of cancer cell chemosensitivity to the hybrid agent (Pearson correlation analysis, *p* < 10^–5^) across a wide range of tissues of origin. The crucial role of hMATE1 was validated in lung adenocarcinoma cells (A549), which expresses high levels of the membrane transporter, using transporter inhibition assays and transient knockdown of the *SLC47A1* gene, in conjunction with quantification of intracellular accumulation of compound **1** and cell viability screening. Preliminary data also show that HCT-116 colon cancer cells, in which hMATE1 is epigenetically repressed, can be sensitized to compound **1** by priming the cells with the drugs EPZ-6438 (tazemetostat) and EED226. Collectively, these results suggest that hMATE1 may have applications as a pan-cancer molecular marker to identify and target tumors that are likely to respond to platinum–acridines.

## Introduction

**Figure 1:**
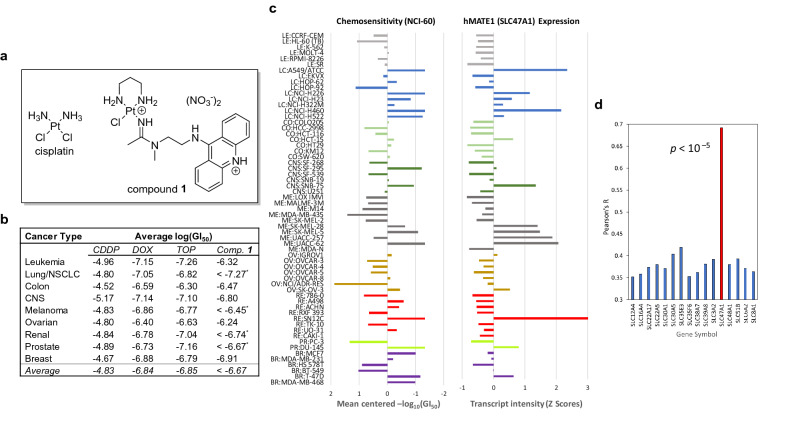
Platinum–acridine agent **1** shows a unique activity profile that correlates with hMATE1 (*SLC47A1*) expression levels in NCI-60 cell lines. (**a)** Structures of cisplatin and hybrid agent **1**. (**b)** Comparative summary of NCI-60 screening results for cisplatin (CDDP), doxorubicin (DOX), topotecan (TOP), and compound **1** based on average growth inhibition (GI_50_ end point, average of 2 assays) for cell lines of different tissues of origin. Asterisks indicate that compound **1** showed cell growth inhibition at log(GI_50_) < – 8 in one or multiple cell lines. A comparison of cell line-specific activity can be found in Supplementary Fig. S1. (**c)** Growth inhibition by compound **1** and *SLC47A1* expression are highly correlated in NCI-60 (mean centered profiles). Left panel: relative sensitivity and resistance to compound **1** at 50% growth inhibition (GI_50_). Right panel: relative transcript intensities (*z*-scores) for *SLC47A1* (*z*-scores for the cell line MDA-MB-468 were not available). Cell lines are color-coded by tissue of origin. (**d)** Correlation of *SLC47A1* expression with chemosensitivity in NCI-60 (Pearson correlation analysis) for positively correlated SLC transporters. A summary of all significantly correlated SLC genes and their (putative) mechanisms of action can be found in Supplementary Table S5.

Since the FDA approval of cisplatin (Fig. 1a), chemically unique approaches have been pursued to improve the efficacy and safety of platinum-based chemotherapy^1^. The design of several of the newer-generation nonclassical metallodrugs is based on the premise that tumor resistance can be overcome at the DNA level as a consequence of the agents’ unique DNA binding modes and DNA damage response (DDR) patterns^2^. This reasoning has redefined the landscape of platinum anticancer drug discovery and resulted in promising new clinical and preclinical candidates^3^. One type of compound in preclinical development are platinum–acridine agents, represented by compound **1** (Fig. 1b), the most potent derivative^4^ identified in this class of cytotoxics^5,6^. Platinum–acridines bind to DNA by a mechanism that involves intercalation and platination of nucleobase nitrogen, causing a more severe form of DNA damage than the cross-links observed for cisplatin^5^. On a per-adduct basis, the hybrid agents are more potent inhibitors of DNA synthesis than cisplatin, which induce replication fork arrest and a high level of DNA double-strand breaks requiring specialized DNA repair modules^7^, and are more efficient transcription inhibitors^8^. These mechanisms most likely contribute to the high cytotoxicity of platinum–acridines, particularly in non-small-cell lung cancer (NSCLC), where the hybrid agents show up to 1,000-fold higher activity than cisplatin^6^. Collectively, the results from mechanistic studies in cell-free systems, human cancer cells, and chemical genomic fitness profiling in *S. Cerevisiae*^9^ are consistent with nuclear DNA as the principal target of these agents.

Platinum–acridines show a dramatically higher activity than cisplatin in NSCLC, even though the hybrid adducts are repaired more rapidly than the classical cross-links in these notoriously DNA repair-proficient cells^7^. These findings call into question whether the damage at the genome level and the cellular response platinum–acridines cause alone overcome chemoresistance in NSCLC. In this article, we report the results of a study that combined activity screening and gene expression correlation analysis, as well as functional target validation performed on compound **1**. We not only demonstrate a complete lack of similarity of the compound’s antitumor profile with that of the classical platinum drugs, but also discovered a membrane transporter, human multidrug and toxin extrusion protein, hMATE1 (*SLC47A1*), as the single most predictive marker of chemosensitivity to platinum–acridines and demonstrate its potential utility as a target for personalized cancer treatment.

## Materials and methods

### Compound screening

Compound** 1** was tested by the NCI Developmental Therapeutics Program in a panel of 59 cancer cell lines in a one-dose screen at 10 µM test compound and in five-dose screens over a concentration range of 10^–4^ to 10^–8^ M. Five-dose screens were performed in duplicate. Reported GI_50_ values and the chemosensitivity profiles (mean graphs) are means of the two experiments. All correlation analyses were based on GI_50_ assay endpoints^10^.

### Correlation and gene set overlap analysis

Comparative analysis of NCI-60 activity profiles based on GI_50_ end points was performed with the COMPARE analysis tools^11^ (dtp.cancer.gov/private-compare) versions 20190306 and 20190828. Both the *Standard Agents* and *Marketed Drugs* databases were searched using GI_50_ values as the endpoint and the following parameters: min. Pearson correlation coefficient, *R* = 0.00; min. number of common cell lines in seed and target vector, 55 or 56; min. standard deviation for seed and target vector, 0.05; number of results, 2000. Correlations between GI_50_ values and gene expression patterns based on transcript levels (*z*-scores) from 5 different microarray platforms were analyzed in a similar manner for a total of 58 cell lines with a minimum correlation of *R* =  ± 0.30 (for *N* = 58, *R* =  ± 0.259 is statistically significant at *p* < 0.05). The CellMiner tool^12,13^ was used to compare the gene expression, DNA copy number alteration, and DNA methylation status for *SLC47A1* across NCI-60 cell lines (database version 2.2, https://discover.nci.nih.gov.cellminer; human genome version HG19, number of genes: 25,683). Correlation analysis of *SLC47A1* transcript levels (average log2 intensities) and DNA methylation (scores 0–1 for completely unmethylated to completely methylated gene promoters) was done with CellMinerCDB (version 1.1; discover.nci.nih.gov/cellminercdb), which implements the GDSC (Sanger Institute) cell line set and databases^12^. Correlations between newly defined gene sets and the MSigDB gene sets encompassing a total of 38,055 genes were calculated using hypergeometric distribution analysis with a false discovery rate q-value < 0.05 (gsea-msigdb.org)^14^.

### Drugs, reagents, siRNA, and antibodies

Compound** 1** was synthesized according to a published procedure^6^ (analytical purity > 97% for NCI-60 and all cell-based assays). All biological assays were performed with appropriately (serially) diluted 10 mM stock solutions of compound **1** in dimethylformamide (DMF). DMF controls were included in all experiments to confirm that the solvent had no effect on cell viability and other assay parameters. The epigenetic drugs, EED226 (HY-101117), tazemetostat (EPZ-6438) (HY-13803), valproic acid (HY-10585) and decitabine (HY-A0004) were purchased from MedChemExpress (Monmouth Junction, NJ, USA). Pyrimethamine (46706), phenazine methosulfate (PMS, P9625), and bovine serum albumin solution (BSA, A8412) were purchased from Sigma Aldrich (St. Louis, MO, USA). MTS reagent was purchased from Promega (G1112) (Madison, WI, USA). RIPA buffer (89901), protease inhibitor mix (87785), and BCA Protein Assay Kit (23227) were purchased from Thermo Fisher (Waltham, MA, USA). Lipofectamine transfection reagent, RNAiMAX, was purchased from Invitrogen (13778100) (Carlsbad, CA, USA). Opti-Mem reduced serum media was purchased from Gibco (31985062) (Gaithersburg, MD, USA). The hMATE1 (*SLC47A1*)-specific pre-designed siRNA and scrambled RNA controls were purchased from Thermo Fisher (Life Sciences Solutions, Carlsbad, CA, USA) (see Supplementary Table S1). Primary and secondary antibodies used for immunoblotting and immunofluorescence applications were purchased from Abcam (Cambridge, MA, USA), Bethyl (Montgomery, TX, USA), and Thermo Fisher/Invitrogen (Waltham, MA, USA) (see Supplementary Table S2 for details of usage).

### LC–MS analysis

The chemical compatibility of pyrimethamine and compound **1** was tested in PBS-buffered solution at 37 °C for 72 h. Prior to LC–MS analysis, buffer salts were removed using Pierce C18 spin columns (Thermo Fisher, Cat. No. 89870) and samples were redissolved in HPLC grade solvent. LC–MS profiles were analyzed on a Bruker Amazon-SL LC–MS system equipped with an electrospray source using an Agilent ZORBAX SB-C18 analytical column (5 mm, 4.6 × 150 mm, PN 883975-902). Pyrimethamine did not undergo undesired ligand substitution chemistry with compound **1** (data not shown).

### General cell culture maintenance

The human cell lines, A549 (lung adenocarcinoma, doubling time 21 h) and HCT-116 (colorectal carcinoma, doubling time 17 h) were obtained from the American Type Culture Collection (ATCC) (Manassas, VA, USA). A549 cells were cultured in DMEM/F12K media (Thermo Fisher, 11330-032) supplemented with 10% FBS (Thermo Fisher, A3160601) and 10% penstrep (Thermo Fisher, 15070-063), unless stated otherwise. HCT-116 cells were cultured in RPMI 1640 (Gibco, A10491-01) with the same additives as above. Cells were incubated at a constant temperature at 37 °C in a humidified atmosphere containing 5% CO_2_ and subcultured every 2–3 days to maintain cells in logarithmic growth. All experiments used cells with passage numbers of less than 20. Cells were tested periodically for mycoplasma infections using Hoechst 33258 DNA staining.

### Uptake of compound 1 studied by confocal fluorescence microscopy

Images were collected on an LSM 880 Confocal Microscope (Carl Zeiss Microscopy) using a 63 × /1.4 NA Plan-Apochromatic objective. To allow comparative fluorescence intensity analysis, excitation power, pinhole settings, PMT gain, and offset values across and within imaging sessions for each respective channel were not changed. Zen software 2.5 (blue edition, Carl Zeiss Microscopy GmbH, 2018) was used for image processing. Panels were assembled and annotated without any additional enhancements of images, unless explicitly stated, in Adobe Photoshop CC, version 2017.1.1. For details of the assays and sample preparations, see the Supplementary Information.

### Uptake of compound 1 studied by ICP-MS

Protocols for the quantification of intracellular platinum–acridines by ICP-MS have been described previously.^15^ Briefly, cells collected from the transporter inhibition and hMATE1 knockdown assays (see below) were pelleted and homogenized by microwave-assisted digestion (ETHOS UP Milestone, Sorisole, Italy) in a mixture of dilute, trace-metal grade HCl and HNO_3_. Standard curves appropriate for quantification of platinum in specified uptake assays were generated using concentrations of 0 ppt, 20 ppt, 50 ppt, 100 ppt, 200 ppt, and 500 ppt of a diluted Pt standard (High-Purity Standards, Charleston, SC, USA). An 8800 Triple Quadrupole ICP-MS spectrometer (Agilent, Tokyo, Japan) equipped with a SPS 4 automatic sampler, a Scott-type double pass spray chamber operated at 2 °C, and a Micromist concentric nebulizer was used for analysis. Helium gas (≥ 99.999% purity, Airgas, Colfax, NC, USA) was used in the collision/reaction cell to minimize potential spectral interferences while monitoring the isotope ^195^Pt. For details of the assays and sample preparations, see the Supplementary Information.

### Cell proliferation assays

The cytotoxicity studies were carried out on nonpyrogenic polystyrene 96-well cell culture plates (Corning Inc., Corning, NY, USA) according to a standard protocol^6^ using the colorimetric Celltiter 96 AQueous Non-Radioactive Cell Proliferation Assay (Promega, Madison, WI, USA). Relative cell viability was determined from the viability of treated and untreated (control) cells. IC_50_ values were calculated from sigmoidal curve fits of log[compound **1**] vs. response in GraphPad Prism 7 (GraphPad Software, San Diego, CA, USA). For the number of replicates and level of significance in each assay, see figure captions in the Results section. Details of the assays and sample preparations are available as Supplementary Information.

### Immunoblotting

Cells were lysed in RIPA buffer (25 mM Tris–HCl pH 7.6, 150 mM NaCl, 1% NP-40, 1% sodium deoxycholate, 0.1% SDS) according to the manufacturer’s protocol. RIPA buffer was supplemented with protease inhibitors. Plated cells were washed twice with ice-cold PBS buffer and then lysed with cold RIPA buffer for 30 min on ice with occasional swirling. Lysed cells were collected with a cell scraper and transferred into a 15 mL microcentrifuge tube. Cell lysates were then sonicated using a Branson Digital Sonifier 450 (settings: 10% pulse, 1 s on/1 s off, for 20 s) and centrifuged at 14,000 × *g* for 15 min at 4 °C. Total protein concentrations were quantified using a BCA Protein Assay Kit.

Protein samples were denatured by incubation in a sample buffer (Thermo Fisher, 39001) supplemented with DTT (50 mM) at 46 °C for 30 min. Equal amounts of total protein were loaded per lane and separated by SDS–polyacrylamide electrophoresis in 4–15% Mini-PROTEAN TGX Precast Protein Gels (Bio-rad, 456-1083) in Tris–glycine SDS buffer (Fisher, BP13414) (30 min at 50 V and 30 min at 120 V). The proteins were wet-transferred to nitrocellulose membranes (Advansta, San Jose, CA, USA, L-08002-010) (2 h at 100 V) (transfer buffer: 25 mM Tris-base, 190 mM glycine, 20% methanol. adjusted to pH 8.3). Membranes were then (i) blocked in TBST buffer (20 mM Tris, 150 mM NaCl and 0.05% Tween 20, adjusted to pH 7.6, 5% non-fat milk) at room temperature for 1 h, (ii) incubated with primary anti-MATE1 antibody or GAPDH antibody in TBST buffer (2% non-fat milk) at 4 °C overnight, (iii) washed 4 times for 5 min with TBST buffer and incubated with goat-anti-rabbit IgG-HRP secondary antibody in TBST buffer (2% non-fat milk) at room temperature for 1 h, (iv) washed with TBST 4 times for 5 min, and (v) finally incubated with SuperSignal West Pico PLUS Chemiluminescent Substrate (Thermo, 34580) at room temperature for 5 min. The protein bands were visualized alongside pre-stained protein ladder (PageRuler, Thermo Fisher) using an Amersham Imager 600 (GE Healthcare). Band intensities were integrated using Image J (version 1.52a, National Institutes of Health, Bethesda, MD). To generate sufficient quantities of cell-free extract for Western blot analysis accompanying knockdown experiments, A549 cells were seeded at a density of 150,000 cells per well on 6-well plates, and transfections were performed with an optimized siRNA concentration of 2.5 nM. Likewise, to quantify hMATE1 (*SLC47A1*) in epigenetic sensitization assays, 100,000 HCT-116 cells were seeded into 60-mm dishes and treated with a 2.5 µM or 5 µM mixture of EPZ-6438 and EED226. Cell lysates were generated in both cases as described above.

### Statistical analysis

Statistical significance of experimental results for two-sample group comparisons was determined with a two-tailed Student t-test. One-way ANOVA with a Bonferroni test and 95% confidence intervals was used for comparisons of three or more sample groups with one independent variable (GraphPad Prism 7, GraphPad Software, San Diego, CA, USA).

## Results and discussion

### Compound 1 shows high potency and a unique activity profile among DNA-targeted anticancer agents

We took advantage of the 60-cell line screen maintained by the Developmental Therapeutics Program (DTP) of the US National Cancer Institute (NCI-60) in combination with the COMPARE analysis tools^11^ to study the biological activity of compound **1** and to assess if mechanistic similarities exist with clinically relevant oncology drugs. Compound **1** was screened twice in a library of 59 cell lines from nine different tissues of origin. The 10 cell lines most sensitive to compound **1** (50% growth inhibition endpoint: logGI_50_ < -7.75, which corresponds to GI_50_ < 18 nM) were NCI-H460, NCI-H226, NCI-H522, and A549 (all NSCLC), SF-295 (glioblastoma), SN12C (renal cell carcinoma), SK-MEL-5 and UACC-62 (both melanoma), DU-145 (prostate), and T-47D (triple-negative breast cancer), representing cancer models from six different tissues of origin and of varying oncogene and tumor suppressor status (Supplementary Table S3). In six of these cell lines (incl. 3 NSCLC), compound **1** resulted in 50% growth inhibition at single-digit nanomolar concentrations (logGI_50_ < − 8) (Supplementary Table S3). Compound **1** showed approximately two orders of magnitude higher activity across the entire spectrum of cell lines than cisplatin, which results in an average growth inhibition similar to that achieved by doxorubicin and topotecan, two oncology drugs also acting through DNA damage-mediated mechanisms (Fig. 1b). While the two topoisomerase poisons kill cancer cells at similar inhibitory concentrations as compound **1**, they do not show the cell line-specific cytotoxic enhancement of our hybrid agent, which is most notable in NSCLC. Of the four agents in comparison, compound **1** shows the widest range of activity from low-nanomolar to micromolar GI_50_ values with a more than 2000-fold difference between the most sensitive and the most resistant cell lines (ΔlogGI_50_ > 3.3, Supplementary Fig. S1).

We then used the COMPARE algorithm in conjunction with Pearson correlation analysis^16^ to search the NCI database for test compounds that resulted in NCI-60 activity patterns similar to that of compound **1**. The results demonstrate that the mechanism of compound **1** is unique among DNA-targeted cytotoxic drugs and other classes of cancer chemotherapeutics (*R* < 0.5) (Table 1). Of the approved oncology drugs tested in NCI-60, transcription inhibitors and topoisomerase poisons revealed the highest similarity with compound **1**. Importantly, cisplatin and oxaliplatin were among the drugs that showed the lowest level of correlation. These results suggest that our hybrid molecule and the traditional platinum-based drugs may not share any relevant mechanistic features except their proven ability to form adducts with nuclear DNA. This raises the question as to what causes the high potency of compound **1** and whether its activity profile might be associated with specific molecular targets or gene expression patterns in cancer cells.

**Table 1:** COMPARE analysis of chemosensitivity profiles for compound **1** and selected anticancer drugs.

Test compound	DNA damage	Mechanism	Pearson’s *R*
Compound 1	Pt-IC hybrid	Inhibitor of DNA synthesis and transcription	1
Mitomycin C	Alk, XL	Inhibitor of rRNA synthesis	0.499*
Doxorubicin	IC	Topo II poison, oxidative stress	0.449
Topotecan	IC	Topo I poison	0.344
Actinomycin D	IC	Transcription inhibitor	0.286
Bleomycin	SC	O_2_-dependent DNA double-strand breaks	0.221*
Erlotinib	N/A	Protein kinase inhibitor	0.187
Gemcitabine	N/A	Inhibitor of DNA synthesis	0.18
Rapamycin	N/A	Inhibitor of mTOR growth signaling	0.123
Paclitaxel (Taxol)	N/A	Microtubule-targeted mitotic inhibitor	0.123*
Cisplatin	Pt, XL	Transcription inhibitor	0.116
Vinblastine	N/A	Microtubule-targeted mitotic inhibitor	0.099*
Oxaliplatin	Pt, XL	Inhibitor of replication and transcription, non-DNA damage mediated mechanisms	0.015

*Pt* platinating agent, *XL* cross-linker, *IC* intercalator, *Alk* alkylating agent, *SC* strand cutter.

Asterisks indicate drugs for which no five-dose NCI-60 data were available in the concentration range − 8 < log[drug] <  − 4 (used for screening compound **1**). In these cases, correlations were based on analysis of alternative concentration ranges for test compounds.

### The chemosensitivity of cancer cells, regardless of tissue of origin, to compound 1 is highly positively correlated with hMATE1 (*SLC47A1*) expression

To gain insight into the factors driving the unique activity profile of compound **1**, a comparative analysis of cell growth inhibition data and global gene expression in NCI-60 cell lines was performed based on gene transcript (mRNA) levels, which are available as part of the COMPARE tools^17,18^. COMPARE analysis yielded 806 unique genes correlated positively, and 849 genes correlated negatively (*p* < 0.05) with the growth inhibition of compound **1** (GI_50_ endpoint) across the entire range of cell lines (Supplementary Table S4). The by far strongest positive correlation (*R* = 0.69, *p* < 10^–5^) was observed with the gene *SLC47A1*, which encodes a member of the solute carrier (SLC) family of proteins: human multidrug and toxin extrusion protein 1, hMATE1. hMATE1, a 13-helix transmembrane protein^19^, shows high expression levels in normal liver and renal tissue (Supplementary Fig. S2), where it serves as a proton-coupled antiporter^20^. Its primary function is the ATP-independent efflux of organic cations across apical membranes into the bile and urine, which renders hMATE1 an essential modulator of drug response, drug toxicity, and drug–drug interactions^21^. Aberrantly high expression of hMATE1 is often also observed in cancer tissue (Supplementary Fig. S2).

The above analysis is consistent with a mechanism by which MATE promotes the uptake of compound **1** into cancer cells rather than acting as an efflux pump (its normal function), which would cause a more resistant phenotype and would have resulted in a negative correlation. A comparison of the NCI-60 screening results for compound **1** with the *SLC47A1* expression profile (Fig. 1c) supports the findings of the COMPARE analysis and illustrates the extent to which the transport protein dominates chemosensitivity. With a few exceptions, cell lines showing high levels of *SLC47A1* transcript are generally exquisitely sensitive to compound **1**, while the opposite is true for cell lines expressing low levels (Fig. 1c). Compound **1** performs poorly relative to other DNA-targeted drugs (e. g., doxorubicin and topotecan, Supplementary Fig. S1) across all leukemia cell lines, which invariably show low *SLC47A1* expression. In cell lines representing solid tumors, considerable cell line-dependent variability exists. For instance, in the two prostate cancer cell lines tested, PC-3 (GI_50_ ≈ 5 µM, low *SLC47A1* expression) and DU-145 (GI_50_ < 10 nM, high *SLC47A1* expression), compound **1** shows a more than 500-fold difference in growth inhibition, which is not observed for any other oncology drug in NCI-60. Likewise, the renal carcinoma cell line, SN12C, which shows the highest level of *SLC47A1* expression of all NCI-60 cell lines, most likely due to a gene copy number amplification^22^ (Supplementary Fig. S3), was also the most sensitive to compound **1**.

*SLC47A1* is not the only solute carrier gene whose expression showed a positive correlation with growth inhibition in NCI-60, but only *SLC47A1* correlated at such a high level (*p* < 10^–5^ vs. *p* < 0.01 for all other SLC genes; see Fig. 1d and Supplementary Table S5), suggesting a specific and dominant role of this transporter in the mechanism of compound **1**. When calculating overlaps between the > 800 genes that were positively correlated with the activity of compound **1** and gene ontology (GO) gene sets deposited in the Molecular Signatures Database (MSigDB^14^), GO terms such as *plasma membrane function and components*, and *intracellular transport* ranked highest (Supplementary Table S6). This is in stark contrast to doxorubicin and topotecan, which showed the greatest overlap with GO sets annotated *chromatin*, *DNA damage recognition and repair*, and *chromosome organization* (data not shown), as would be expected for a genotoxic agent^9^. These observations underpin the notion that, contrary to our expectation, the chemosensitivity of cancer cells to compound **1** is not controlled at the genome level, but by the transportome.

### Pyrimethamine, a selective hMATE1 inhibitor, effectively blocks the cellular accumulation of compound 1 and quenches its cytotoxicity in A549 cells

**Figure 2:**
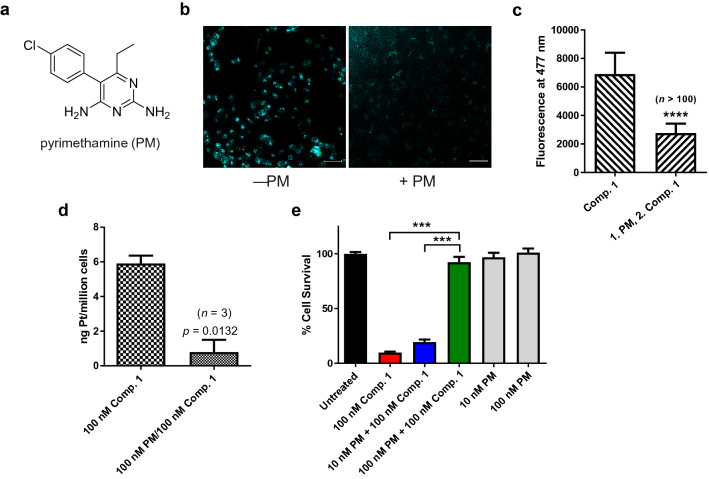
Pyrimethamine (PM) protects A549 lung adenocarcinoma cells from the cytotoxic effects of compound **1** by blocking its cellular uptake. (**a)** PM, a high-affinity, selective inhibitor of hMATE1. (**b)** Confocal fluorescence microscopy images of A549 cells treated for 4 h with 10 µM compound **1** with or without PM. Scale bars: 20 µm. Acridine fluorescence in the blue channel is displayed in cyan. (**c)** Mean fluorescence intensities in the acridine channel (arbitrary units) of > 100 selected A549 cells (treated according to the conditions in panel **(b)** determined in 6 images from 2 independent experiments; ****, *p* < 10^–4^, mean ± S.D., two-tailed t-test with unequal variance. (**d)** Accumulation of compound **1** (100 nM, 4 h) in A549 cells in the absence and presence of PM (100 nM) determined by inductively coupled mass spectrometry, ICP-MS), data are the mean of three independent experiments ± S.E.M, two-tailed t-test. (**e)** Cytoprotective effect of PM-mediated inhibition of uptake of compound **1** into A549 cells monitored by a cell proliferation assay. Data are the mean of two experiments performed in sextuplicate (*n* = 12) ± S.E.M; ***, *p* < 0.001, one-way ANOVA with post hoc test.

To validate hMATE1 protein as a mediator of chemosensitivity, we first performed a transporter inhibition assay in A549 human lung adenocarcinoma cells. A549 expresses high levels of hMATE1 (*SLC47A1*) (The Human Genome Database; see Supplementary Fig. S2), which we confirmed by Western blot analysis (Supplementary Fig. S4). Unsurprisingly, the cell line proved to be highly sensitive to compound **1** in the NCI-60 screen (GI_50_ < 10 nM) and in previous colorimetric cell proliferation assays (IC_50_ = 3.9 nM)^4^. In this assay, prior to treatment with compound **1**, cultured A549 cells were pre-treated with the antimalarial drug pyrimethamine (PM, Fig. 2a), a potent and selective inhibitor of hMATE1 (reported *K*_i_ values: 77–93 nM^23^). Since the assay required co-incubation of compound **1** and PM, we first confirmed that no undesired reactivity exists between the two agents. When A549 cells were pre-treated with PM, followed by a 4-h exposure to compound **1**, confocal microscopy images showed a reduction of intracellular acridine fluorescence by 60% relative to cells not treated with PM (Fig. 2b,c). These results suggest that hMATE1-mediated transport across the plasma membrane is directly involved in the cellular uptake of compound **1**.

Because the microscopy experiments were performed at relatively high concentrations of platinum–acridine and PM (10 µM), contributions from non-specific transport by other membrane proteins cannot be ruled out under these conditions^24^. To overcome this drawback, we took advantage of the parts-per-trillion-level limit of detection of inductively coupled plasma mass spectrometry (ICP-MS) and also quantified uptake of compound **1** from cellular platinum levels under therapeutically more relevant conditions. When cells were pre-incubated with 100 nM PM to avoid non-specific inhibition of other organic cation transporters and subsequently treated with 100 nM compound **1**, corresponding to the compound’s IC_90_ value in A549, a decrease of uptake by 85% was observed (Fig. 2d). Together, these findings corroborate that compound **1** is selectively transported across the plasma membrane by hMATE1.

To determine if blocking hMATE1 by PM had an effect on the cytotoxicity of compound **1** in A549 cells, we performed a colorimetric cell proliferation assay (Fig. 2e). Exposure to 100 nM compound **1** for 72 h causes severe cell death with less than 10% of the cells surviving treatment. When A549 cells were pre-treated with PM at concentrations that did not compromise cell viability, a pronounced cytoprotective effect was observed. PM at a concentration of 10 nM was able to increase the population of viable cells to 20%, while 100 nM inhibitor resulted in 90% survival (*p* < 0.001) of cells treated with compound **1**. The level of protection achieved at the latter concentration of PM correlates well with the reduced platinum levels determined by ICP-MS (Fig. 2d), providing additional support for the notion that hMATE1-mediated transport is the key to compound **1**’s high potency.

### Gene knockdown by RNA interference (RNAi) further validates the role of hMATE1 protein in the mechanism of compound 1

**Figure 3:**
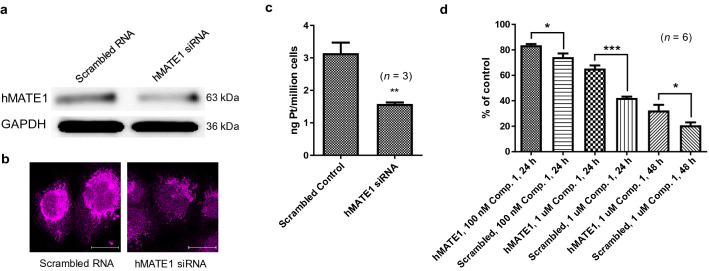
Transient knockdown of the membrane transporter hMATE1 (*SLC47A1*) attenuates uptake and cytotoxicity of compound **1**. (**a**) Western blot analysis of hMATE1 and GAPDH (loading control) protein levels in A549 cells reverse-transfected with scrambled RNA sequence (“mock”) (left) or hMATE1 siRNA (right) (one 72-h transfection at 2.5 nM siRNA). Full-length blots are presented in Supplementary Fig. S9. (**b**) Immunofluorescence staining of fixed, permeabilized A549 cells 72 h after siRNA knockdown or mock treatment. Scale bars: 20 µm. (**c**) Uptake of compound **1** into A549 cells after siRNA or mock transfection determined by ICP-MS. Accumulated platinum (ng/10^6^ cells) is shown as the mean ± S.E.M. of three independent experiments. The assay was performed several times under slightly varied conditions with similar results (see Supplementary Fig. S5); *p* < 0.01, **). (**d**) Effect of hMATE1 knockdown on the cytotoxicity of compound **1** in A549 cells assessed by a cell proliferation assay (MTS). Data are the mean ± S.E.M of two independent experiments performed in triplicate (*n* = 6; the results were significant at *p* < 0.05 (*) and *p* < 0.001 (***), respectively; two-tailed t-test). For additional data and replicates, see Supplementary Fig. S5.

Ultimate evidence for a direct role of hMATE1 transporter in promoting the cellular accumulation and cytotoxicity of compound **1** came from gene knockdown experiments using RNA interference (RNAi). Such an assay is complicated by the non-trivial task of combining transient gene silencing with a long-term cell proliferation assay. Using transfection of appropriate siRNAs, we were able to generate a A549 model in which hMATE1 was transiently reduced by 40–50% relative to scrambled control, which is consistent with reported knockdown efficiencies achieved for the *SLC47A1* gene in this cells line using RNAi^25^. Knockdown was confirmed by Western blot analysis and immunofluorescence intensity evaluation of transfected cells (Fig. 3a,b). The cellular uptake of compound **1** was studied under the same conditions as in the transporter inhibition assay using PM. In hMATE1 knockdown cells, accumulation of platinum was significantly (*p* = 0.0091) reduced by 50% relative to control cells transfected with a scrambled RNA sequence (Fig. 3c). We then designed a 96-well plate assay that allowed us to assess the performance of compound **1** in A549 cells after hMATE1 knockdown (Fig. 3d). After 24 h of continuous treatment, the dose- and time-dependent cytotoxicity of compound **1** was reduced in A549 cells at concentrations of 100 nM and 1 µM by 12% and 35%, respectively. At the higher concentration, the level of protection persists after 48 h of treatment, which resulted in a 36% higher survival of hMATE1-silenced cells compared to mock-treated cells. These results unequivocally confirm that hMATE1 protein plays a direct role in the mechanism of compound **1** by mediating its cellular uptake, which ultimately controls the chemosensitivity of the lung cancer cell line.

### Transcriptomics and gene set overlap analysis suggest that hMATE1 expression is epigenetically regulated in many types of cancer

Pattern comparisons in NCI CellMiner reveal that significant correlations exist between *SLC47A1* transcript levels and DNA methylation status (CpG islands, CGI) of the gene (*p* < 0.001), as well as correlations involving epigenetic repressors of gene expression, such as DNA methyltransferase I (DNMT1) and the histone methyltransferase, enhancer of zeste homolog 2 (EZH2) (Tables S7 and S9). Thus, in addition to DNA copy number amplifications (Supplementary Fig. S3, Supplementary Table S7), epigenetic alterations appear to dominate hMATE1 expression in cancer tissue. This was also confirmed in an extended set of 963 cell lines in the Genomics of Drug Sensitivity in Cancer database (GDSC, Sanger Institute)^26^ for which *SLC47A1* expression is strongly negatively correlated with CGI methylation (Pearson’s *R* = − 0.32, *p* = 4.9 × 10^–25^) (Supplementary Fig. S6, Supplementary Table S8). Recent studies of HCT-116 colorectal cancer cells (see below) and normal human liver tissue, in which hMATE1 expression is attenuated epigenetically by promoter hypermethylation^27^, support the results of the correlation analyses.

In NCI-60 cell lines, more than 400 genes (CellMiner) were identified whose methylation status is negatively correlated with *SLC47A1* transcript levels, including *SLC47A1* itself (Tables S5 and S9). When we performed a gene set overlap analysis on this a priori defined gene set^14^ with gene sets in the Molecular Signatures Database (GSEA, MSigDB, gsea-msigdb.org), the highest correlation was observed with genes epigenetically silenced in embryonic stem cells (Supplementary Table S10)^28^. Silencing of the latter genes involves EZH2-mediated histone protein H3 trimethylation at lysine 27 (H3K27me3) by the polycomb repressive complex 2 (PRC2) and downstream promoter CGI hypermethylation. These observations provide additional clues about hMATE1 regulation at the epigenome level, which led us to hypothesize that epigenetic drugs reversing repression of hMATE1 might lead to an increase in cellular uptake of compound **1** and sensitize resistant cancer cells to this agent.

### Treatment of HCT-116 colon cancer cells with epigenetic drugs activates hMATE1 expression and enhances the cellular uptake and cytotoxicity of compound 1

To test if cancer cells can be sensitized to compound **1** by priming with epigenetic drugs, we chose the colon cancer cell line HCT-116. HCT-116 cells show low hMATE1 expression caused by repressive modifications in its *SLC47A1* promoter region (see Supplementary Fig. S6) and proved to be relatively resistant to compound **1** in NCI-60 (Fig. 1c, Supplementary Fig. S1).

**Figure 4:**
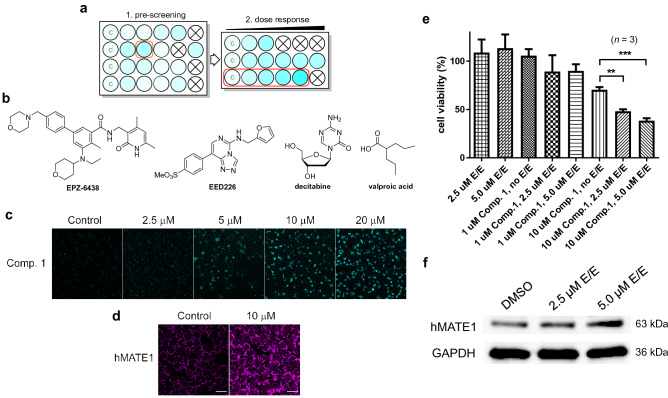
Epigenetic, PRC2-targeted drugs sensitize HCT-116 colon cancer cells to compound **1**. (**a)** Schematic layout of drug screening assay. A darker cyan color indicates higher levels of drug accumulation based on fluorescence intensity. Wells labeled ‘C’ are no-treatment controls (DMSO) and crossed-out wells indicate tested concentrations of drug affect cell viability. (**b)** Structures of epigenetic drugs used in this assay. (**c)** Microscopy images of HCT-116 cells exposed to 10 µM compound **1** for 4 h after pretreatment with varying concentrations of EPZ-6438 and EED226 (“E/E”) for 72 h. Scale bars: 20 µm. (**d)** Immunofluorescence staining of cells from the control and 10 µM treatment groups Scale bars: 20 µm. (**e)** Viability of HCT-116 cells pre-treated with epigenetic drugs determined using cell proliferation assays (MTS). Data are presented as the mean ± S.E.M. for an assay performed with triplicate wells (**, *p* < 0.01; ***, *p* < 0.001; one-way ANOVA with post hoc test). For replicates of this assay, see Supplementary Fig. S8B. (**f**) Expression levels of hMATE1 and GAPDH (loading control) in HCT-116 cells determined by Western blot analysis under the same conditions as described in panel **(e)**. Full-length blots are presented in Supplementary Fig. S10.

We first pre-screened several epigenetic drugs in cultured HCT-116 cells in a multi-well plate format for their ability to increase the uptake of compound **1** using fluorescence microscopy (Fig. 4a, see caption for conditions). Cells were treated with four epigenetic drugs that are currently being studied in advanced phase clinical trials: EPZ-6438 (tazemetostat, a potent inhibitor of enhancer of zeste homolog 2, EZH2)^29^, EED226 (an allosteric inhibitor of the polycomb repressive complex 2, PRC2)^30,31^, decitabine (a DNA methyltransferase I, DNMT1, inhibitor)^32^, and valproic acid (a histone deacetylase, HDAC, inhibitor)^33^, as well as combinations of these drugs (Fig. 4b). Epigenetic drugs have previously been demonstrated to enhance the expression of epigenetically silenced genes in HCT-116^34^, including the *SLC47A1 *gene^35, 36^. EPZ-6438 and EED226, alone or in combination, resulted in enhanced uptake of compound **1**, based on the observation of increased acridine-associated, blue fluorescence in the confocal microscopy images, without causing changes in cell morphology and viability (Supplementary Fig. S7). These compounds were then tested again at escalating doses (2.5–20 μM) (Fig. 4a). A combination of EPZ-6438 and EED226 (“E/E”) resulted in the most pronounced increase in uptake of compound **1** in a dose-dependent manner (Fig. 4c). Additionally, images of representative cells stained with hMATE1 antibody showed a higher level of immunofluorescence compared to the no-treatment control, which was considered preliminary evidence of increased hMATE1 expression (Fig. 4d).

We then used a cell proliferation assay to determine if pre-exposing HCT-116 cells to non-toxic concentrations of EPZ-6438 and EED226 sensitized them to compound **1**. At higher concentrations, the epigenetic drugs alone also caused significant changes in the cells’ growth characteristics and significant cell death. Because of this limitation, the experiments were only performed with 2.5 µM and 5 µM E/E. When cells were treated with 10 µM compound **1**, pre-exposure to E/E resulted in a pronounced decrease in cell viability that was dependent on the dose of epigenetic drug. At 5.0 µM E/E, the maximum enhancement in cell growth inhibition relative to unsensitized control was 45% (Fig. 4e). Under these ad hoc conditions, Western blot analysis of lysates from HCT-116 cells show a 20% and 70% increase in hMATE1 levels relative to control at the lower and the higher concentration, respectively (Fig. 4f). This observation in conjunction with the microscopy results (Fig. 4c,d) strongly suggests that hMATE1 protein is the mediator of the chemosensitizing effect. The results of this proof-of-concept experiment demonstrate the feasibility of sensitizing cancer cells to compound **1** using nontoxic concentrations of epigenetic drugs.

At physiological pH, compound **1** and its derivatives exist as 2 + charged, hydrophilic cations comprising a positively charged platinum(II) moiety and a protonated 9-aminoacridine chromophore (p*K*_a_ = 9–10)^15^. In earlier work, we have demonstrated that the most potent platinum-acridines accumulate in NSCLC cells at a 60–100-fold faster rate than cisplatin^37^, which is consistent with the efficient, SLC transporter-mediated uptake mechanism established in this study. Compound **1** is the first chemotherapeutic agent for which bioinformatics and high-throughput screening tools have identified an overexpressed transport protein as a target that confers a high level of chemosensitivity to cancer cells.

Compound **1** has emerged from a pipeline of platinum*–*acridine agents that were designed based on the guiding principle that rapid formation of unique DNA adducts would overcome tumor resistance to DNA-targeted drugs, including platinum-based pharmaceuticals. While DNA damage indisputably is the ultimate cause of cancer cell death produced by the hybrid agent, its low-nanomolar activity critically depends on a transport protein, which is an unprecedented feature among anticancer drugs in the NCI-60 database. hMATE1 controls the pattern of activity with a high level of predictability. Cancer cells overexpressing the membrane transporter are highly sensitive to compound **1** regardless of genetic background and phenotypic abnormalities^38,39^. This is an important observation since efficient transmembrane transport that leads to high intracellular drug concentrations has the potential to overcome common resistance mechanisms such as DNA repair^40^ or multidrug resistance-mediated drug efflux^41^.

hMATE1 expression is high in most NSCLC cell lines (Fig. 1c), which explains why the advantage of platinum–acridines over cisplatin and other cytotoxic agents was first noted in this aggressive form of cancer^5^. Membrane transporters that help drugs accumulate in diseased tissue may ultimately result in a more favorable therapeutic window for systemic treatment^42^. Compound **1** has already demonstrated efficacy in xenograft models of A549 in mice when administered intravenously, both directly and as liposomal formulation^43^. Using a non-optimized dosing schedule, the agent was able to reduce tumor growth by 65% with less than 20% weight loss in test animals, which was reversible, without causing other signs of systemic toxicity. It is possible that hMATE1-enhanced uptake into tumors contributes to the efficacy of compound **1** in vivo.

A few cases have been reported of membrane transporters typically involved in drug elimination that may also enhance drug uptake into tumor tissue. Organic cation transporters (hOCT, *SLC22A*) are an example of such a dual pharmacokinetic role^44^. hOCTs have been shown to enhance the cytotoxicity and efficacy of platinum-containing drugs^42,45^. For instance, in colorectal cancer tissue, high levels of hOCT assist in the cellular uptake of oxaliplatin, which has provided a rationale for the drug’s therapeutic use in this form of cancer^46^. hMATE1 protein, which mediates efflux of substrate from polarized epithelial cells in excretory organs, may play a similar role by transporting substrates across the plasma membrane into cells^20^. This has recently been demonstrated for the clinical kinase inhibitor imatinib (Gleevec) in chronic myeloid leukemia (CML) cells, which enhances the drug’s potency in this hematological cancer^47^. Importantly, in the same study hMATE1 expression levels have been validated as a predictor of interindividual differences in imatinib response and clinical outcome in CML patients^47^. These findings corroborate the critical role solute carrier (SLC) transporters may play in mediating delivery of pharmacologically relevant levels of drug to diseased tissue^48^.

Finally, we provide proof-of-concept data to demonstrate that colorectal cancer cells treated with epigenetic drugs can be sensitized to compound **1** and that the enhanced cytotoxicity is caused by hMATE1-mediated drug accumulation. A growing number of clinical and preclinical studies support the utility of co-administering cytotoxic drugs with epigenetic drugs (see also clinicaltrials.gov). Liu et al.^49^ recently demonstrated that renal cell carcinoma (RCC) cells can be sensitized to oxaliplatin by pre-treatment with the hypomethylating agent decitabine, which promotes hOCT2 expression and oxaliplatin accumulation. Another compelling case of epigenetic sensitization has been reported by Gardner et al.^50^ for the Schlafen-11 protein (*SLFN11*), a putative RNA/DNA helicase that acts as a sensor of replicative stress and tumor suppressor^51^. In patient-derived small-cell lung cancer (SCLC) tissue, Schlafen-11, which sensitizes cancer cells to topoisomerase I poisons, was epigenetically silenced^50^. Treatment with epigenetic drugs restores Schlafen-11 levels, which reverses resistance in SCLC and re-sensitizes cells to the drug topotecan^50^. There also appears to be an epigenetic component to hMATE1 (*SLC47A1*) expression in SCLC^52^ (sclccelllines.cancer.gov). Since topotecan is a substrate of hMATE1^53^, the reported level of sensitization to the topoisomerase I poison in SCLC cell lines after treatment with EPZ-6438^51^ may also reflect higher drug accumulation due to increased levels of hMATE1. Using compound **1** as a cytotoxic component in similar combination regimens to treat SCLC and other cancers not responding optimally to our hybrid agent (e.g., leukemias, colorectal cancer, ovarian cancer, see Fig. 1), would be an attractive opportunity.

## Conclusion

In summary, the current study provides the mechanistic basis for the unique spectrum of anticancer activity of a platinum–acridine hybrid agent, compound **1**. The data demonstrates that the fate of a cancer cell treated with compound **1** is decided at the plasma membrane. The results underscore the crucial role of hMATE1 in mediating intracellular delivery of oncology drugs and as a potential pan-cancer marker of drug responsiveness. In addition, epigenetic priming may present a new strategy for tackling intractable tumors with platinum–acridines and other oncology drugs targeting this membrane transporter. These features render compound **1** a unique cytotoxic agent, which may have applications as a component of personalized combination regimens to treat resistant tumors.

## Supplementary information

Supplementary Information 1.

Supplementary Information 2.

Supplementary Table S7.
