# Supplementary material for: A membrane transporter determines the spectrum of activity of a potent platinum–acridine hybrid anticancer agent
Source: Sci Rep. 2020 Sep 16;10:15201. doi: 10.1038/s41598-020-72099-z (PMC7494928; doi:10.1038/s41598-020-72099-z)
Supplement: Supplementary file 2 — Supplementary Information 2. [file 41598_2020_72099_MOESM2_ESM.pdf]

Supplementary Information

for

A Membrane Transporter Determines the Spectrum of Activity of a Potent Platinum–Acridine Hybrid Anticancer Agent

Xiyuan Yao, Noah H. Watkins, Heather Brown-Harding, and Ulrich Bierbach✉

✉ bierbau@wfu.edu

Table of Contents **page**

Table S1. Summary of RNA sequences used in RNAi experiments **S3**

Table S2. Summary of antibodies used in assays **S4**

Table S3. Ten NCI-60 cell lines most sensitive to compound **1**/genetic backgrounds **S5**

Figure S1. Comparison of NCI-60 chemosensitivity profiles **S6**

Table S4. Results of NCI COMPARE analysis **S7**

Table S5. NCI-60/COMPARE analysis/summary of SLC genes **S29**

Figure S2. Expression of hMATE1 (*SLC47A1*) in normal human tissue and in cancer cells **S30**

Figure S3. *SLC47A1* gene copy numbers and transcript levels in NCI-60 **S31**

Table S6. Summary of top 10 gene set overlaps in MSigDB for compound **1** **S32**

Figure S4. Western blot analysis of A549 cell lysate for hMATE1 expression **S34**

Figure S5. (A) Western blot for optimized RNAi conditions. (B) hMATE1 antibodies used in this study and aligned amino-acid sequences of the 63-kDa full-length protein and the 34-kDa splice variant (NCBI). (C,D) Results for drug uptake experiments after hMATE1 knockdown **S35**

Table S7. Pattern comparisons for *SLC47A1* expression in NCI CellMiner **S36**

Figure S6. Correlation between CPI methylation status and expression levels of *SLC47A1* **S37**

Table S8. Summary of significant ($p < 0.05$) correlations identified between CPI methylation status and expression levels of the *SLC47A1* gene **S38**

Table S9. Summary of correlations for chemosensitivity and omics data for compound **1** **S39**

Table S10. Summary of top 10 overlaps between all gene sets of the molecular signatures database (MSigDB) and the input gene set of hypermethylated genes negatively correlated with *SLC47A1* expression. **S40**

Figure S7. Pre-screening of HCT-116 colon cancer cells for the effects of epigenetic drugs on cell viability and accumulation of compound **1**. **S42**

Figure S8. (A) Western blot analysis of hMATE1 protein in HCT-116 cell lysates. B) Cell proliferation assay for HCT-116 cells. **S43**

Figure S9. Full-length, uncropped/unprocessed image of Western blots presented in Fig. 3a **S44**

Figure S10. Full-length, uncropped/unprocessed image of Western blots presented in Fig. 4f **S45**

Assay Layouts AL1–AL4 **S46**

Experimental Details for Assays____________________________ **S48**

Assays and sample preparation for confocal microscopy analysis_____________ **S48**

Assays and sample preparation for ICP-MS analysis__________________ **S49**

Assays and sample preparation for cell viability analysis________________ **S49**

SI References____________________________________**S51**

Table S1. Summary of RNA sequences used in RNAi experiments.

	Seq	ID[a]	Sense (5´to 3´)	Antisense (5´to 3´)
**I**	Silencer siRNA	140539	CCGAGACAUCAUUAAUCUGtt	CAGAUUAAUGAUGUCUCGGtc
**II**	Silencer Select siRNA1	s30533	CAAACUUGAUUUCCCAGUAtt	UACUGGGAAAUCAAGUUUGcc
**III**	Silencer Select siRNA2	s30534	GAUCGUAACUGGAGUUGCAtt	UGCAACUCCAGUUACGAUCt
**IV**	Silencer Negative Control #3 siRNA	AM4615	N/A	N/A
**V**	Silencer Select Negative Control #1 siRNA	4390843	N/A	N/A

[a] All Thermo Fisher.

Table S2. Summary of antibodies used in assays.

	**Protein**	**ID**	**Application**	**Dilution**
**1**	anti-MATE1 (*SLC47A1*) antibody	ab104016[a]	immunoblotting	1:1,000
**2**	anti-GAPDH antibody	A300-639A-M[b]	immunoblotting	1:1,000
**3**	goat-anti-rabbit IgG-HRP secondary antibody	G-21234[c]	immunoblotting	1:10,000
**4**	anti-hMATE1 (*SLC47A1*) antibody	PA5-2527[c]	immunofluorescence	1:300
**5**	goat-anti-rabbit IgG Alexa Fluor-635 secondary antibody	A-31576[c]	immunofluorescence	1:400

[a] Abcam, [b] Bethyl, [c] Thermo Fisher/Invitrogen.

Table S3. Ten NCI-60 cell lines most sensitive to compound **1** and their genetic backgrounds

			Mutated Cancer Genes						
**Tissue of Origin**	**Cell Line**	**$log_{10}GI_{50}$**	***CDKN2A***	***TP53***	***PTEN***	***RB1***	***PIK3CA***	***KRAS***	***BRAF***
Lung	NCI-H460	< -8.00	🟥				🟦	🟥	
	NCI-H226	< -8.00	🟥						
	NCI-H522	-7.92		🟥					
	A549	< -8.00	🟥					🟥	
CNS	SF-295	-7.88	🟥	🟥	🟥				
Renal	SN12C	< -8.00		🟥					
Melanoma	SK-MEL-5	-7.75	🟥						🟦
	UACC-62	< -8.00	🟥		🟥				🟥
Prostate	DU-145	< -8.00	🟥	🟦		🟥			
Breast	T-47D	-7.83		🟥			🟦		

🟥 Homozygous mutation/deletion 🟦 Heterozygous mutation/deletion (based on refs. 1 and 2)

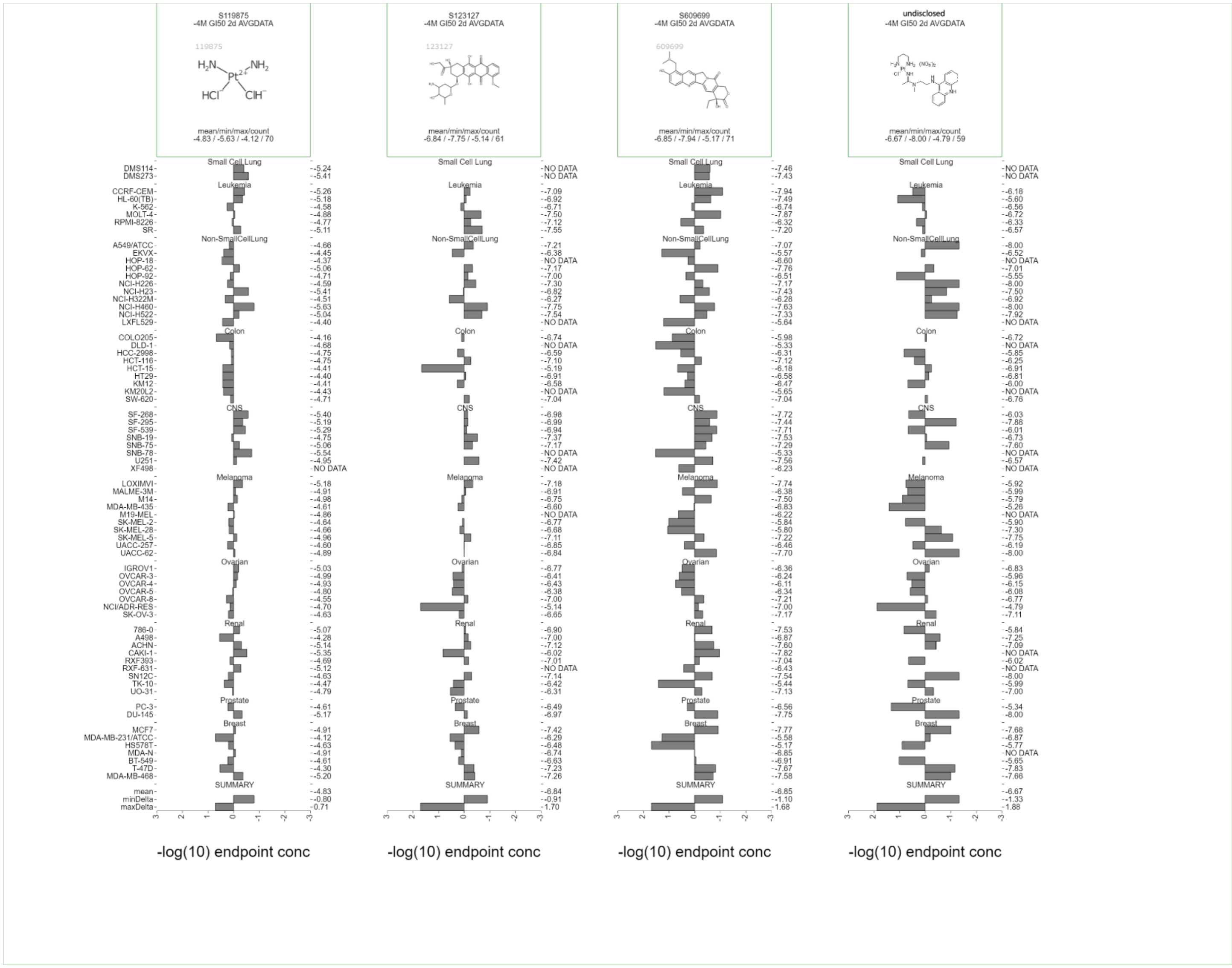

Figure S1. Comparison of NCI-60 chemosensitivity profiles (averages of at least 2 assays) for cisplatin (CDDP, NSC 119875), doxorubicin (DOX, NSC 123127), topotecan (TOP, NSC 609699), and compound **1** (NSC # not disclosed).

Table S4. Results of NCI COMPARE analysis: positive and negative correlations between chemosensitivity (NCI-60, logGI50) and microarray gene expression data (z-scores)

Entry	Positive Correlation		Negative Correlation	
#	Pearson's *R*	Gene Symbol	Pearson's *R*	Gene Symbol
1	**0.692**	**SLC47A1**	-0.525	ZNF330
2	0.541	CCDC104	-0.52	CCT8
3	0.535	FAM120AOS	-0.511	MYSM1
4	0.533	CCDC113	-0.504	TMEM222
5	0.531	LOC100268168	-0.501	CIDEB
6	0.527	DOLK	-0.489	MRPL41
7	0.522	NQO1	-0.485	RQCD1
8	0.519	POLDIP2	-0.477	ADCY10P1
9	0.513	ARL8A	-0.477	DUSP7
10	0.509	PHLPP2	-0.476	WRAP73
11	0.508	MOCS2	-0.475	GK2
12	0.499	LOC100287590	-0.474	NDST1
13	0.497	DOCK1	-0.474	SMU1
14	0.495	AKAP10	-0.473	PEX5
15	0.495	TNPO1	-0.47	DR1
16	0.489	LOC728431	-0.47	FOXJ3
17	0.487	ULK1	-0.47	RFX3
18	0.486	ZNF652	-0.469	LARS
19	0.484	TMTC3	-0.468	WASF2
20	0.478	CLIP1	-0.466	SYNPO2
21	0.476	TENC1	-0.462	SYNCRIP
22	0.476	KIAA1841	-0.461	SMIM8
23	0.476	C5AR1	-0.453	ZNF451
24	0.475	SQSTM1	-0.451	MAP3K19
25	0.474	TMEM218	-0.449	RBM15
26	0.474	LOC100287525	-0.447	KHSRP
27	0.473	ANKRD40	-0.446	LRRC3B
28	0.47	SNRNP27	-0.446	PVT1
29	0.468	TACO1	-0.444	ADH1B
30	0.467	FBXL20	-0.441	CSRNP3
31	0.465	IFT27	-0.44	IPO5
32	0.464	ZNF219	-0.44	DNAH2
33	0.464	UNC119	-0.44	CSF2
34	0.463	HFE	-0.44	DPH2
35	0.463	PSMD11	-0.438	MKI67IP

	$p < 0.00001$	*****
	$p < 0.0001$	****
	$p < 0.001$	***
	$p < 0.01$	**
	$p < 0.05$	*

36	0.462	TAOK1	-0.438	TARP
37	0.462	SEMA3C	-0.435	BABAM1
38	0.459	SKP1	-0.434	NCS1
39	0.457	TXNRD1	-0.434	EXOSC3
40	0.456	TMLHE	-0.433	FAM186B
41	0.455	LRRC23	-0.433	DIAPH2
42	0.454	HSF4	-0.431	EPAG
43	0.453	ZBTB40	-0.431	VCPIP1
44	0.453	MAN1A2	-0.43	FAM91A1
45	0.452	ADSSL1	-0.428	TTYH1
46	0.452	ACACB	-0.428	RBM33
47	0.452	PCGF2	-0.428	TTTY9B
48	0.451	BCAS4	-0.427	SLC24A2
49	0.451	TOB1	-0.427	BTF3L4
50	0.45	RBPMS	-0.425	TNFRSF10C
51	0.449	SNAPIN	-0.425	LOC100129250
52	0.449	RNPEP	-0.424	ST6GAL1
53	0.445	PTGR1	-0.424	JPH3
54	0.443	NINJ1	-0.424	TFAP4
55	0.443	SRXN1	-0.422	ZNF473
56	0.443	NFYB	-0.421	FHIT
57	0.441	RHBDL3	-0.421	XAB2
58	0.439	WBP1	-0.421	ZMYM2
59	0.437	PSMD3	-0.42	VAPB
60	0.437	NT5C3B	-0.42	KCNQ5
61	0.437	STAT3	-0.42	ZNF800
62	0.437	FBXW4	-0.418	WDR52
63	0.435	COPRS	-0.416	KRT77
64	0.434	FAM188B	-0.416	ZRANB2
65	0.434	F2RL2	-0.416	CCL16
66	0.434	GOLGA1	-0.416	LOC284998
67	0.433	VEZT	-0.415	SLC43A3
68	0.433	ZBTB41	-0.415	POLR1E
69	0.433	NFU1	-0.415	CDKN3
70	0.432	MFSD5	-0.415	KIAA1009
71	0.431	RUFY2	-0.414	RIN3
72	0.431	GABARAPL2	-0.414	TPPP2
73	0.43	TMEM5	-0.414	ORMDL1
74	0.43	OLFM1	-0.414	CHRAC1
75	0.43	TRNP1	-0.414	CCDC18

76	0.43	PQLC3	-0.414	RPA2
77	0.43	HOXC13	-0.414	NKTR
78	0.43	ABHD15	-0.413	FMNL3
79	0.429	TRIM23	-0.413	ADAM1A
80	0.429	ZNF268	-0.413	AKAP8
81	0.429	FBXO21	-0.413	TMED5
82	0.429	HOXA-AS2	-0.412	UBE2V2
83	0.428	KIAA0319	-0.412	LOC643837
84	0.428	KYNU	-0.412	ATXN3
85	0.428	TBK1	-0.412	INPP4B
86	0.428	TMEM132A	-0.412	NMUR1
87	0.428	MPP3	-0.411	ZZZ3
88	0.427	BLVRA	-0.41	TRMT61A
89	0.427	EGF	-0.41	LPCAT4
90	0.427	IRX2	-0.41	U2AF1
91	0.427	WIPF2	-0.41	PPP1R8
92	0.426	RHBDD2	-0.409	C4orf47
93	0.425	SETD1A	-0.409	ATAD3A
94	0.425	UNKL	-0.409	ELL
95	0.425	FDXR	-0.409	TMEM69
96	0.424	G6PD	-0.409	LINC00337
97	0.424	LETM1	-0.408	LYAR
98	0.424	NLGN4Y	-0.407	PDE4C
99	0.424	WISP2	-0.406	LRRC58
100	0.423	MMP28	-0.406	ODF2L
101	0.422	TUBG1	-0.406	RPF2
102	0.422	MTA3	-0.406	TWF2
103	0.421	ERAL1	-0.406	ZNF251
104	0.421	MED13	-0.406	USE1
105	0.421	LRIG2	-0.405	OTUD6B
106	0.421	VMO1	-0.405	TCEB1
107	0.42	DAZAP2	-0.405	HIST1H4D
108	0.42	ATXN1L	-0.405	PDIA5
109	0.42	JOSD2	-0.404	MDM2
110	0.42	AP1G1	-0.404	SMARCA4
111	0.419	IDNK	-0.403	MUC3
112	0.419	SPATS2	-0.402	MBD1
113	0.419	SLC35E3	-0.402	UBE3D
114	0.419	CD163	-0.402	ANKZF1
115	0.419	DYNC2LI1	-0.402	GSTM5

116	0.419	TLE2	-0.4	C19orf70
117	0.419	MAP2K4	-0.399	CCNB1IP1
118	0.419	VMP1	-0.398	DBT
119	0.418	GATA5	-0.397	LYNX1
120	0.418	TP53I11	-0.397	DHRS4-AS1
121	0.418	SDF2	-0.397	DNAJA1
122	0.417	FBXO15	-0.397	SLC22A7
123	0.417	PCDHA	-0.397	STX12
124	0.417	AGGF1	-0.397	AGFG2
125	0.417	CDK3	-0.396	CSPG4
126	0.417	NR1H2	-0.396	PLVAP
127	0.416	KIAA0100	-0.396	PTER
128	0.416	TSSK6	-0.396	CEP152
129	0.416	TRADD	-0.395	ZER1
130	0.415	MARK3	-0.393	DDX18
131	0.415	MSL1	-0.393	LINC00312
132	0.415	GSR	-0.393	SMIM12
133	0.415	FAM134B	-0.393	NDUFB6
134	0.414	ATG10	-0.393	PFKFB1
135	0.414	MLIP	-0.393	PLAG1
136	0.413	GJC2	-0.392	SRSF3
137	0.412	CCDC121	-0.391	GRK1
138	0.411	XYLT2	-0.391	LOC100505964
139	0.411	MEGF9	-0.391	HEMK1
140	0.411	FLJ12120	-0.391	NES
141	0.411	SLC30A5	-0.391	OBP2B
142	0.41	TRIM16	-0.391	ZMYND11
143	0.41	AMFR	-0.39	DDX49
144	0.41	SPAG5	-0.39	PHIP
145	0.409	FAM155B	-0.389	MPPE1
146	0.409	SRGAP3	-0.389	PREP
147	0.409	MOB3B	-0.389	GATA5
148	0.409	SNF8	-0.389	ZNF544
149	0.408	RABGAP1	-0.389	E4F1
150	0.408	BLVRB	-0.389	RIOK3
151	0.408	CLTC	-0.388	SCN2B
152	0.407	NKIRAS2	-0.388	LRRC14
153	0.407	SIRT3	-0.388	MON1A
154	0.406	BMPR1A	-0.388	CCDC58
155	0.406	LOC399884	-0.387	METTL8

156	0.406	PSMG4	-0.387	TMEM51-AS1
157	0.405	BLOC1S2	-0.387	ANPEP
158	0.405	IDE	-0.387	APPL1
159	0.405	DHX29	-0.386	PPP1R3E
160	0.404	FAM45A	-0.386	SIRT6
161	0.404	C7orf25	-0.386	WDR27
162	0.404	ANKRD32	-0.386	RASSF1
163	0.404	S1PR3	-0.386	KCNJ4
164	0.403	ARHGEF26	-0.385	UFM1
165	0.403	ADORA3	-0.385	FAM184B
166	0.402	C8orf42	-0.385	ACOT11
167	0.402	RNASEH1	-0.385	RPF1
168	0.402	AMER2	-0.384	SAMD8
169	0.402	SREBF1	-0.384	WDR48
170	0.402	PIGS	-0.383	GTF2B
171	0.401	THSD4	-0.383	GTPBP5
172	0.401	SH3BGR	-0.382	PDE12
173	0.401	LRRC46	-0.382	ATP11C
174	0.401	FAM218A	-0.382	MTUS2
175	0.4	MIEN1	-0.382	TSPO
176	0.4	DLGAP1	-0.382	CLCN6
177	0.4	KCNA1	-0.382	ARHGAP27
178	0.4	ACP1	-0.381	PMAIP1
179	0.399	TVP23B	-0.381	VPS13A
180	0.399	ABHD1	-0.381	GRPEL2
181	0.399	ZNF821	-0.381	CLECL1
182	0.399	EIF2B4	-0.381	LOC100505519
183	0.399	GCLM	-0.381	PAQR3
184	0.398	C2CD3	-0.38	CD22
185	0.398	SMIM20	-0.38	SLC6A19
186	0.397	ZNF132	-0.379	IDI2
187	0.397	NBR1	-0.379	LSM11
188	0.397	AQP11	-0.379	GTPBP1
189	0.397	ATP2A2	-0.379	CELF6
190	0.397	SAR1B	-0.379	SFPQ
191	0.397	RUNDC1	-0.379	LAMTOR3
192	0.396	LOC100132004	-0.379	LPAR1
193	0.396	STX17	-0.379	POGLUT1
194	0.396	TMEM178B	-0.379	PLA2G2E
195	0.396	FAM212B	-0.378	CYB561D2

196	0.396	MEG3	-0.378	AURKAIP1
197	0.395	ASXL2	-0.378	MEX3C
198	0.395	SDSL	-0.378	PHF7
199	0.395	EID3	-0.378	DFFB
200	0.395	PTRH2	-0.378	LOC100499489
201	0.394	PBX1	-0.378	RCL1
202	0.394	PDE4D	-0.378	DNMT1
203	0.394	NCOA7	-0.378	GALNT15
204	0.394	GBF1	-0.378	FLJ39639
205	0.393	MYBL1	-0.378	HLA-DQB1
206	0.393	TAF15	-0.378	RNGTT
207	0.393	SLC51B	-0.377	UMPS
208	0.393	LOC100506603	-0.377	TICRR
209	0.392	C5AR2	-0.377	BRWD3
210	0.392	KDM6A	-0.377	MAX
211	0.392	TMEM106C	-0.376	SRR
212	0.392	LOC100507501	-0.376	LONRF3
213	0.392	GFM1	-0.376	SMARCB1
214	0.392	EML2	-0.376	LHPP
215	0.392	SLC3A2	-0.376	C8orf82
216	0.391	JAM3	-0.376	BCL10
217	0.391	FBF1	-0.375	LOC100505697
218	0.391	RNF40	-0.375	PNMA3
219	0.39	FUNDC1	-0.375	ZNF41
220	0.39	RNF135	-0.375	IQCB1
221	0.39	NALCN	-0.375	POP1
222	0.39	LOC100506668	-0.375	GPBP1L1
223	0.39	IL6ST	-0.375	AZI2
224	0.39	SIDT2	-0.375	TFB2M
225	0.39	ZDHHC16	-0.374	KLHL21
226	0.39	PIGX	-0.374	FAM154A
227	0.389	SPR	-0.374	GTPBP8
228	0.389	FKBP15	-0.374	RCC1
229	0.389	IFT43	-0.374	CNTROB
230	0.389	KIAA0195	-0.374	ZADH2
231	0.389	LOC100505564	-0.374	ZNF496
232	0.388	LIX1L	-0.374	SLC2A9
233	0.387	MARCO	-0.374	AGXT
234	0.387	BRCA1	-0.373	RTFDC1
235	0.387	GNRHR	-0.373	OPRL1

236	0.387	BAD	-0.373	MTO1
237	0.387	EPHX1	-0.373	TGS1
238	0.386	EXOC6B	-0.373	EIF3G
239	0.386	EPDR1	-0.373	JRK
240	0.386	PPP1R9A	-0.373	MRPL34
241	0.386	CCDC127	-0.373	TWISTNB
242	0.386	APH1B	-0.373	DDX28
243	0.386	PHF12	-0.372	ZNF259
244	0.385	FAM66D	-0.372	CENPW
245	0.385	ZNF287	-0.372	C8orf33
246	0.385	ZNF688	-0.372	DRD3
247	0.385	JAKMIP3	-0.372	BTLA
248	0.385	C1orf115	-0.372	TCF20
249	0.385	TMED2	-0.372	RAD21
250	0.384	LOC100653206	-0.371	MAN1A2
251	0.384	TRAF4	-0.371	CLK1
252	0.384	HRAS	-0.371	PRKACA
253	0.384	AGBL2	-0.371	TIMM44
254	0.384	FMO5	-0.371	TNN
255	0.383	WDR34	-0.371	FGF22
256	0.383	TUSC3	-0.371	SHOX2
257	0.383	TSKU	-0.37	RNF126
258	0.383	SDK2	-0.37	GRIN1
259	0.383	KDM5B	-0.37	C1orf228
260	0.382	ZMIZ2	-0.37	STOML1
261	0.382	PDCD2	-0.37	BOP1
262	0.382	COX15	-0.37	SPATA8
263	0.382	MYO19	-0.37	LRRC4B
264	0.381	SLC39A8	-0.369	CAAP1
265	0.381	DDB2	-0.369	SEMA6D
266	0.381	IFT140	-0.369	CCNC
267	0.381	RPGRIP1L	-0.369	WWP2
268	0.381	SHC1	-0.369	LONP1
269	0.381	TBC1D5	-0.369	PYGB
270	0.38	SLC22A5	-0.368	CHERP
271	0.38	PCYOX1	-0.368	USP6
272	0.38	ZNF334	-0.368	KCTD5
273	0.38	DHX40	-0.368	AFF2
274	0.38	SLC48A1	-0.368	KDM4D
275	0.38	MAFK	-0.368	PASD1

276	0.38	ZCRB1	-0.367	MRTO4
277	0.38	CHRM4	-0.367	MYOT
278	0.38	BTBD11	-0.367	CBX2
279	0.38	ABHD4	-0.367	PADI4
280	0.38	FGD6	-0.366	LPCAT2
281	0.379	OAZ3	-0.366	SLC17A9
282	0.379	INVS	-0.366	WDTC1
283	0.379	TMEM18	-0.366	MPEG1
284	0.379	LOC339803	-0.365	ZRANB2-AS1
285	0.379	MDM2	-0.365	NDUFB7
286	0.379	DICER1	-0.365	TMEM39A
287	0.379	KIAA0895	-0.365	ENTPD8
288	0.378	LOC100130502	-0.365	ECHDC1
289	0.378	P2RX4	-0.365	ZNF182
290	0.378	DALRD3	-0.365	IGH
291	0.378	ZFAND4	-0.364	TRMT1
292	0.378	ADIPOR1	-0.364	JMJD1C
293	0.378	CUZD1	-0.364	LINC00338
294	0.377	G6PC3	-0.364	OPTC
295	0.377	RNH1	-0.364	SYNJ2
296	0.377	ASPN	-0.364	RAD54L
297	0.377	TMEM256	-0.364	OSGEP
298	0.377	KLF7	-0.364	ROBO4
299	0.377	RDM1	-0.364	TPD52L2
300	0.377	JKAMP	-0.364	TAOK3
301	0.377	CCND3	-0.364	PLD4
302	0.377	RPL3L	-0.363	RPS28
303	0.377	ALPK1	-0.363	ADO
304	0.377	TMEM254-AS1	-0.363	MAP2K7
305	0.376	KRIT1	-0.363	MAP3K7
306	0.376	RAB5C	-0.363	CMKLR1
307	0.376	KBTBD4	-0.363	GANC
308	0.376	SNX17	-0.363	IL11RA
309	0.376	XIAP	-0.363	AQP8
310	0.376	CCS	-0.363	RAB3IP
311	0.375	GPC1	-0.363	NHP2L1
312	0.375	CLU	-0.362	CLPP
313	0.375	ALDH3A2	-0.362	CCR3
314	0.375	NF1	-0.362	DSEL
315	0.375	TP53I3	-0.362	CDC40

316	0.375	DLGAP1-AS1	-0.362	FAM69A
317	0.374	KAT2A	-0.362	UBIAD1
318	0.374	COPZ1	-0.362	EEF1A1
319	0.374	LOC100996321	-0.362	KRTAP10-12
320	0.374	SLC22A17	-0.361	CCDC93
321	0.373	EFNA3	-0.361	TTK
322	0.373	EMX2	-0.361	LOC390705
323	0.373	YLPM1	-0.361	IL12RB1
324	0.373	BTD	-0.361	INTS1
325	0.373	PRDM15	-0.361	PTPN22
326	0.373	IL20RB	-0.361	LOC100499227
327	0.373	MCPH1	-0.361	LOC100240728
328	0.372	CCNB1	-0.361	DPH3
329	0.372	C12orf10	-0.361	MED30
330	0.372	AMZ2	-0.361	KLC4
331	0.372	CISD3	-0.361	DUSP15
332	0.371	KCTD20	-0.36	SRSF11
333	0.371	HCP5	-0.36	SLC26A8
334	0.371	SLC6A2	-0.36	USP37
335	0.371	SETDB1	-0.36	WNT10A
336	0.371	TRIM3	-0.36	BRD4
337	0.371	SLC30A1	-0.36	MIOX
338	0.371	LOC728012	-0.36	CDC37
339	0.37	SHC2	-0.36	N4BP1
340	0.37	EFEMP1	-0.359	POLR2K
341	0.37	BAZ2A	-0.359	C9orf123
342	0.37	RP9P	-0.359	TSHZ1
343	0.37	COQ6	-0.359	IPCEF1
344	0.37	AP3M2	-0.359	ABHD14A
345	0.37	LOC100288911	-0.359	LRIG1
346	0.37	DCST2	-0.358	SCTR
347	0.37	FAM24B	-0.358	GPATCH2L
348	0.369	ZNF687	-0.358	RRS1
349	0.369	BPTF	-0.358	MARK1
350	0.369	FOXN3-AS1	-0.358	SASS6
351	0.369	CSPG5	-0.358	C17orf105
352	0.369	VPS9D1	-0.358	CFC1B
353	0.369	MALSU1	-0.358	RPS6KA1
354	0.369	DND1	-0.357	PLA2G12B
355	0.368	HSPB8	-0.357	MTSS1

356	0.368	ZNF76	-0.357	LINC00852
357	0.368	PAQR6	-0.357	FAM3C
358	0.368	ZDHHC11	-0.357	TSPAN11
359	0.368	AGFG1	-0.357	LOC100508120
360	0.368	NR5A2	-0.357	ERO1L
361	0.368	ALG13	-0.356	RPS6KA2
362	0.368	ETAA1	-0.356	PRAC
363	0.367	HSPB7	-0.356	MAT1A
364	0.367	SYT3	-0.356	DSCR3
365	0.367	PLEKHH3	-0.356	MYL7
366	0.367	SIAH1	-0.356	NOM1
367	0.367	FMNL3	-0.356	HOXA-AS4
368	0.367	KRT86	-0.356	SERBP1
369	0.367	TXNRD3	-0.356	HYAL3
370	0.366	TNRC18	-0.356	GNL2
371	0.366	PYY	-0.355	ILVBL
372	0.366	LXN	-0.355	USP33
373	0.366	NEO1	-0.355	SERP1
374	0.366	SIX5	-0.355	CYTH3
375	0.366	LYRM9	-0.354	PAPSS1
376	0.366	DHX57	-0.354	PGPEP1
377	0.365	MFF	-0.354	TSGA10
378	0.365	MAPT	-0.354	PRPF38A
379	0.365	SDCBP2-AS1	-0.354	UBE3A
380	0.365	C2orf76	-0.353	CCNJ
381	0.365	NPPA	-0.353	LOC285812
382	0.365	LYPLAL1	-0.353	UHRF2
383	0.364	MTMR12	-0.353	AK2
384	0.364	ULK2	-0.353	FBXO10
385	0.364	COL4A4	-0.353	NSUN6
386	0.364	CD99P1	-0.353	SPEN
387	0.364	ARL2BP	-0.353	SRSF12
388	0.364	STX6	-0.353	TRPM1
389	0.364	SMIM14	-0.352	EPHA6
390	0.364	RAB4B	-0.352	SLC27A4
391	0.364	LEPREL4	-0.352	FOSL2
392	0.364	CWC25	-0.352	PPP3CC
393	0.364	SLC8A1	-0.352	BLK
394	0.364	HP07349	-0.352	LRRC47
395	0.364	PSMD4	-0.352	AZIN1

396	0.364	FAM192A	-0.352	FOXK2
397	0.363	KIAA1324	-0.352	PLTP
398	0.363	PSMA2	-0.352	NCSTN
399	0.363	SIX3-AS1	-0.352	DCLRE1C
400	0.363	ZMAT5	-0.352	C3orf38
401	0.363	ASMT	-0.352	TMEM133
402	0.363	TOP2A	-0.352	LRRC40
403	0.362	DDX52	-0.352	PFDN6
404	0.362	AMACR	-0.352	DIS3
405	0.362	STAMBP	-0.352	HIVEP3
406	0.362	SLC38A7	-0.352	DCN
407	0.362	ARF3	-0.352	SREBF2
408	0.362	METRN	-0.352	FAM182B
409	0.362	DCAF8	-0.351	BPTF
410	0.362	GLI4	-0.351	NUP214
411	0.362	ST3GAL2	-0.351	ZNF710
412	0.361	CCL26	-0.351	TMEM110
413	0.361	SNHG10	-0.351	SYNPO2L
414	0.361	CHD9	-0.351	DSG3
415	0.361	ZNF577	-0.351	DNAH7
416	0.361	OMD	-0.351	TRPV4
417	0.36	TOM1L2	-0.351	TMEM207
418	0.36	GRIP1	-0.351	ARID1A
419	0.36	ERLEC1	-0.351	C1orf86
420	0.36	EP400	-0.351	ZNF324B
421	0.36	CFHR4	-0.35	TACO1
422	0.36	GALNT8	-0.35	ANKRD13C
423	0.36	PRPSAP2	-0.35	ZNF292
424	0.36	UBE3B	-0.35	PPIP5K2
425	0.359	ATRAID	-0.35	CCDC130
426	0.359	ADAM17	-0.35	DOK3
427	0.359	MBD5	-0.35	VPS13D
428	0.359	EHD3	-0.35	DESI2
429	0.359	RARB	-0.35	LOC221814
430	0.359	LOC149134	-0.35	ETS1
431	0.359	SURF1	-0.35	HSF1
432	0.358	GFM2	-0.35	TBC1D26
433	0.358	APLP1	-0.35	SREK1
434	0.358	FAM76A	-0.35	RAC2
435	0.358	TAX1BP1	-0.349	PIGK

436	0.358	LINC00669	-0.349	NPC1
437	0.358	NRBP1	-0.349	RGCC
438	0.358	SLC16A4	-0.349	PGS1
439	0.358	ZNF385A	-0.349	CLEC12A
440	0.358	CACNG4	-0.349	MAPKAPK3
441	0.358	B4GALNT1	-0.349	C18orf54
442	0.358	LOC100507481	-0.349	GSPT1
443	0.358	ZNF341	-0.349	TIMM13
444	0.358	PIP4K2B	-0.349	FLG-AS1
445	0.358	SERF1B	-0.349	BZRAP1
446	0.357	CTSL1	-0.349	GOLGA8A
447	0.357	CCDC19	-0.349	MMP9
448	0.357	IFT20	-0.349	SHANK3
449	0.357	SPPL2A	-0.348	PITHD1
450	0.357	THNSL2	-0.348	RREB1
451	0.357	NFIC	-0.348	APBA1
452	0.357	BAIAP2-AS1	-0.348	TAF4B
453	0.357	METAP1	-0.348	HCG18
454	0.357	FTO	-0.348	CREBZF
455	0.357	ZNF281	-0.348	EIF5B
456	0.356	TSKS	-0.347	OGFOD3
457	0.356	FAM224B	-0.347	GRIK1-AS1
458	0.356	C5orf51	-0.347	MLLT1
459	0.356	POMZP3	-0.347	SMAD2
460	0.356	ALOXE3	-0.347	TOE1
461	0.355	ORMDL2	-0.347	NKX2-1-AS1
462	0.355	PAGR1	-0.347	CCL23
463	0.355	ZFPM1	-0.347	TRIM25
464	0.355	LHFPL4	-0.347	GNL1
465	0.355	SLK	-0.347	RGS13
466	0.355	MIF4GD	-0.347	CDK6
467	0.355	RND1	-0.347	PAK1IP1
468	0.354	TLCD1	-0.347	CEP63
469	0.354	TMOD3	-0.347	GART
470	0.354	PEX13	-0.346	C2orf69
471	0.354	AQP8	-0.346	CDKN2B
472	0.354	PLEKHA2	-0.346	HSPA1L
473	0.354	SIX3	-0.346	CBLC
474	0.354	TMEM37	-0.346	SMARCC1
475	0.354	TRIP11	-0.346	SPIB

476	0.354	ZNF839	-0.346	FASTKD5
477	0.354	WASL	-0.346	LOC100131825
478	0.354	SMARCC2	-0.346	TRMT13
479	0.353	WAC	-0.346	FCAR
480	0.353	CDK5RAP2	-0.346	SNHG6
481	0.353	NOL3	-0.346	ZNF644
482	0.353	PPP2R2C	-0.346	LNX2
483	0.353	SUB1	-0.346	RTCA
484	0.353	ME1	-0.345	LINC00293
485	0.353	TBC1D9B	-0.345	STAB2
486	0.353	FRA10AC1	-0.345	CCAR1
487	0.353	ZNF140	-0.345	ZNF775
488	0.353	HRH3	-0.345	VSTM2A
489	0.353	SPATA20	-0.345	FARSA
490	0.353	SLC35F6	-0.345	DRD2
491	0.353	GADD45G	-0.345	TOMM22
492	0.353	FLJ39632	-0.345	WDR90
493	0.353	INSM1	-0.345	PGK1
494	0.352	OSGIN2	-0.345	KCNK7
495	0.352	FGD4	-0.344	SLC16A1
496	0.352	SLC12A4	-0.344	GTF2A1
497	0.352	CACNA2D3	-0.344	FOXO3
498	0.352	NKAP	-0.344	MAFF
499	0.352	SMO	-0.344	LOC200609
500	0.352	CIDEC	-0.344	CCNY
501	0.352	TRIM68	-0.344	NECAP2
502	0.351	C1orf56	-0.344	PSMG1
503	0.351	CENPK	-0.344	MPRIP
504	0.351	HIST1H3E	-0.344	ATF6B
505	0.351	LOC100507424	-0.344	TMEM174
506	0.351	GSE1	-0.344	MIR155HG
507	0.351	DCC	-0.343	UBALD2
508	0.351	CAP2	-0.343	ALS2CR12
509	0.351	GNB5	-0.343	PSIP1
510	0.35	SNX29	-0.343	SLCO4A1
511	0.35	COASY	-0.343	SLC7A1
512	0.35	AGBL5	-0.343	SELK
513	0.35	VPS72	-0.343	TES
514	0.35	TCTN1	-0.343	HS3ST6
515	0.35	ARID4B	-0.343	PRKCB

516	0.35	IARS2	-0.343	SUPT16H
517	0.35	SPIRE2	-0.343	MAEL
518	0.35	HIBADH	-0.343	PPM1A
519	0.35	TPD52L1	-0.343	C11orf21
520	0.35	AADAC	-0.343	MYL3
521	0.35	OSMR	-0.342	LOC100996665
522	0.349	DNAJC7	-0.342	C22orf31
523	0.349	GGNBP2	-0.342	SMIM10
524	0.349	FAM134C	-0.342	ELAVL1
525	0.349	TSACC	-0.342	SLC19A1
526	0.349	HDGFRP3	-0.342	FOXN3
527	0.349	SNCB	-0.342	ZNF148
528	0.349	IGSF9B	-0.342	PVR
529	0.349	GAST	-0.342	NUDC
530	0.349	GRAMD1B	-0.342	LOC155060
531	0.349	ADCY6	-0.341	IL6ST
532	0.348	MAGED4	-0.341	ARMC6
533	0.348	HIP1	-0.341	NDRG3
534	0.348	PCGF5	-0.341	COL8A2
535	0.348	LIN7B	-0.341	ZNF503-AS2
536	0.348	NLN	-0.341	FAF1
537	0.348	GAPVD1	-0.341	UROS
538	0.348	GTF3C1	-0.34	LILRB3
539	0.348	CCKBR	-0.34	OXSR1
540	0.348	PLAC4	-0.34	UBE2L6
541	0.348	RAB1A	-0.34	EIF2S3
542	0.348	ZFP1	-0.34	DST
543	0.348	PBXIP1	-0.34	ZNHIT6
544	0.348	DPF1	-0.34	SLC30A7
545	0.347	IGHMBP2	-0.34	RPL5
546	0.347	TMEM107	-0.34	PYDC1
547	0.347	IAH1	-0.34	SLC25A32
548	0.347	TVP23C	-0.34	NPFF
549	0.347	RHBDL1	-0.34	PARP8
550	0.347	GIP	-0.34	ABCA9
551	0.347	PDPN	-0.339	ZEB2
552	0.347	MBTPS1	-0.339	PPIE
553	0.347	PTGES	-0.339	UBAP2L
554	0.347	REEP5	-0.339	SSU72
555	0.347	GPR25	-0.339	MGC12488

556	0.346	CSNK1D	-0.339	TRAF3IP2-AS1
557	0.346	PCGF1	-0.339	ING4
558	0.346	TBCD	-0.339	CLASP2
559	0.346	DNAJC27	-0.339	FOSL1
560	0.346	MAPK9	-0.339	C1orf21
561	0.346	MRPL19	-0.339	GRPEL1
562	0.346	C11orf92	-0.338	RPS11
563	0.346	DYNLL1	-0.338	RGL1
564	0.346	STRN	-0.338	PIK3R5
565	0.345	NDRG4	-0.338	RFX1
566	0.345	AKT1	-0.338	ALDOB
567	0.345	BAI2	-0.338	EPS15
568	0.345	XPR1	-0.338	LZTFL1
569	0.345	C5orf15	-0.338	KCNQ3
570	0.345	PTH1R	-0.338	ANKS6
571	0.345	RFX3	-0.338	RIOK2
572	0.345	ABAT	-0.337	MIIP
573	0.345	CAMSAP1	-0.337	NODAL
574	0.344	ZFP64	-0.337	RASL12
575	0.344	ANKEF1	-0.337	NTSR2
576	0.344	MTX1	-0.337	USP4
577	0.344	SUPT6H	-0.337	SNHG12
578	0.344	TEKT4P2	-0.336	IFI44L
579	0.344	MIER3	-0.336	LOC100505702
580	0.344	ANKRD55	-0.336	KCNC4
581	0.344	DSEL	-0.336	YIPF7
582	0.344	GRAMD1C	-0.336	SELRC1
583	0.344	COL4A6	-0.336	MAK16
584	0.344	UBE2K	-0.336	PROL1
585	0.344	C10orf35	-0.336	SRSF4
586	0.343	ABCC1	-0.336	GRIA1
587	0.343	MYH8	-0.336	SNHG5
588	0.343	GDF11	-0.335	POLR1B
589	0.343	FBXO4	-0.335	UBE4B
590	0.343	ZNF234	-0.335	PIGG
591	0.343	CREBZF	-0.335	ZBTB1
592	0.343	CEP112	-0.335	DGCR2
593	0.343	B3GALT5	-0.335	KLF6
594	0.343	NENF	-0.335	ITGB2
595	0.343	CDKL1	-0.335	DNTTIP2

596	0.342	LRRC16B	-0.335	IRX2
597	0.342	SWI5	-0.335	CNTN5
598	0.342	DUS3L	-0.335	DBR1
599	0.342	KRT10	-0.335	ADAM7
600	0.342	KCNF1	-0.335	TXNL4A
601	0.342	UNC79	-0.335	PURB
602	0.342	DNAH1	-0.335	TMEM68
603	0.342	TP53I13	-0.335	ZNRD1-AS1
604	0.342	MPHOSPH9	-0.335	SCLT1
605	0.342	POLR2J4	-0.335	IGF1
606	0.342	RNF187	-0.335	SAFB
607	0.342	C9orf16	-0.335	LOC283143
608	0.342	SMEK2	-0.335	PCK2
609	0.341	LMTK2	-0.334	HNMT
610	0.341	UBB	-0.334	MDN1
611	0.341	DSN1	-0.334	RGS7BP
612	0.341	LRP10	-0.334	GSDMD
613	0.341	IDH1-AS1	-0.334	ATF6
614	0.341	PBLD	-0.334	SIGLEC6
615	0.341	YTHDC2	-0.334	NUS1
616	0.34	C11orf16	-0.334	ZNF460
617	0.34	LOC100505622	-0.334	OGT
618	0.34	CNTN1	-0.334	HAUS6
619	0.34	CABYR	-0.334	NOTCH4
620	0.34	SOBP	-0.334	CPPED1
621	0.34	TMEM231	-0.334	SERPINB8
622	0.34	LONP2	-0.334	PVRL3
623	0.34	SF3B3	-0.334	RARS2
624	0.34	TMEM245	-0.333	GORASP2
625	0.34	GPR137	-0.333	TSN
626	0.34	CRLS1	-0.333	TBL1XR1
627	0.34	DNAJC28	-0.333	SLC26A6
628	0.34	DHX8	-0.333	ALOX12
629	0.339	ARFGEF2	-0.333	DNAJC8
630	0.339	ABCA8	-0.333	NUPL2
631	0.339	PDCL2	-0.333	UGT2B15
632	0.339	GFOD2	-0.333	OSCAR
633	0.339	RASGEF1C	-0.333	CNPPD1
634	0.339	PXN	-0.333	NOP16
635	0.339	C1orf27	-0.333	LOC401176

636	0.339	RECQL5	-0.333	MUL1
637	0.339	TCEA2	-0.333	MIR143HG
638	0.339	ODF3L1	-0.332	KCNK10
639	0.339	MON2	-0.332	ZNF266
640	0.339	ZNF226	-0.332	FUS
641	0.338	CDK12	-0.332	ATP10A
642	0.338	GTF2H2	-0.332	TPSG1
643	0.338	ATP6V0A1	-0.332	NCAPH2
644	0.338	FGG	-0.332	CSPP1
645	0.338	AK7	-0.332	PPIL3
646	0.338	DPY19L1P1	-0.332	KRTDAP
647	0.338	TFE3	-0.332	AMD1
648	0.338	TMEM8B	-0.332	STK17B
649	0.338	GAA	-0.332	PARL
650	0.338	CHD3	-0.332	HLF
651	0.338	C17orf97	-0.331	RORA
652	0.338	ZDHHC12	-0.331	ADRA1A
653	0.338	CYB5D2	-0.331	TLE4
654	0.337	NSF	-0.331	DIO2
655	0.337	JAK3	-0.331	MTFR1
656	0.337	TUBD1	-0.331	CHD4
657	0.337	PI4KB	-0.331	DCAF13
658	0.337	TAF10	-0.331	GNG5
659	0.337	RNF112	-0.331	ABCB1
660	0.337	FCER1A	-0.331	WNK2
661	0.337	IGFBP4	-0.331	RAB8A
662	0.337	KCNK15	-0.331	YME1L1
663	0.337	CD160	-0.331	ZBTB7A
664	0.337	NES	-0.331	TSC2
665	0.337	PPAP2A	-0.331	ZFPL1
666	0.337	SULT4A1	-0.331	OSBPL2
667	0.337	MTFMT	-0.33	ZRANB1
668	0.337	AP4E1	-0.33	NIPAL3
669	0.337	C2orf72	-0.33	CHRM2
670	0.337	TRPV5	-0.33	DOCK8
671	0.337	ITLN1	-0.33	FAR2
672	0.336	LLGL1	-0.33	ZNF445
673	0.336	ASTN2	-0.33	CDX4
674	0.336	COX7A2L	-0.33	EMILIN2
675	0.336	CACNA1D	-0.33	RD3

676	0.336	AKT1S1	-0.33	DNASE1L1
677	0.336	PTPLAD1	-0.33	SNRPE
678	0.336	ZNF609	-0.33	STX4
679	0.336	FAM177A1	-0.33	LOC100996694
680	0.336	MFI2-AS1	-0.329	KCNQ2
681	0.336	CCDC74A	-0.329	ISY1
682	0.336	MAR6	-0.329	LOC146880
683	0.336	WWC2-AS2	-0.329	REG3A
684	0.336	ACBD6	-0.329	UTP23
685	0.336	GOSR1	-0.329	C15orf39
686	0.336	VPS41	-0.329	SSSCA1
687	0.336	SULT1A3	-0.329	SDR16C5
688	0.336	AP3B1	-0.329	CHD7
689	0.336	CUEDC1	-0.329	ARID1B
690	0.335	AP2B1	-0.329	PRCD
691	0.335	TBX3	-0.329	WDR81
692	0.335	USP9X	-0.329	RANGAP1
693	0.335	ZNF223	-0.328	CDC5L
694	0.335	TCTN3	-0.328	TBC1D25
695	0.335	SMC2	-0.328	OBSL1
696	0.335	COQ9	-0.328	DNAJC9
697	0.335	DHH	-0.328	CNOT8
698	0.335	LINC00674	-0.328	WNT6
699	0.334	SMKR1	-0.328	TTTY7
700	0.334	FLOT2	-0.328	KPNA1
701	0.334	KIF3A	-0.328	GATAD2A
702	0.334	SULT1A2	-0.328	CCDC86
703	0.334	YIPF4	-0.327	BMP8B
704	0.334	TEF	-0.327	PPP2R3C
705	0.334	ADCY1	-0.327	TFB1M
706	0.334	ZNF711	-0.327	TRIM27
707	0.334	LRRC37A	-0.327	IFNK
708	0.334	SYNGR4	-0.327	CMSS1
709	0.334	TRIM32	-0.327	NCAN
710	0.334	RNF103	-0.327	CDRT15L2
711	0.334	LOC100506746	-0.327	YPEL2
712	0.334	CLPTM1L	-0.327	DHX30
713	0.334	LOC101060027	-0.327	HOXB-AS5
714	0.334	FAM102A	-0.327	WBSCR27
715	0.334	ZFR2	-0.326	OBP2A

716	0.333	TTTY2	-0.326	MRPL3
717	0.333	THSD7A	-0.326	IL9
718	0.333	ACLY	-0.326	QRSL1
719	0.333	LOC100505938	-0.326	ZNF430
720	0.333	EGFL7	-0.326	LARP1B
721	0.333	MTX3	-0.326	IGHG1
722	0.333	ABCB6	-0.326	LOC101060009
723	0.333	RAB3C	-0.326	CCNT2
724	0.333	SKI	-0.326	SEP15
725	0.333	METTL9	-0.326	HMGA1
726	0.333	ZNF638	-0.326	RAB27A
727	0.333	NUP98	-0.326	PDX1
728	0.332	AKR1C2	-0.326	DENND2A
729	0.332	C1QTNF3	-0.326	GCSHP3
730	0.332	IFI6	-0.326	TEX12
731	0.332	PAIP1	-0.326	AP2B1
732	0.332	DAW1	-0.325	PITPNB
733	0.332	H2AFY2	-0.325	PTGER1
734	0.332	ALDH7A1	-0.325	FBXL6
735	0.332	PMS2P3	-0.325	PDLIM5
736	0.332	KPNB1	-0.325	STX3
737	0.332	CLDN8	-0.325	EBNA1BP2
738	0.332	GLI1	-0.325	HSPA8
739	0.332	B9D1	-0.325	GMPR2
740	0.331	RSBN1L	-0.325	UGT8
741	0.331	SCN8A	-0.325	RNPS1
742	0.331	GKAP1	-0.325	RPL23AP7
743	0.331	ZNF701	-0.325	RSF1
744	0.331	MST1	-0.325	SNX31
745	0.331	HTRA1	-0.325	OPRM1
746	0.331	TREM1	-0.324	HIPK2
747	0.331	LOC100129935	-0.324	ESRRA
748	0.331	LOC100507367	-0.324	FBXO41
749	0.33	AKIP1	-0.324	TEKT2
750	0.33	LINC00473	-0.324	ZMYM6
751	0.33	USP42	-0.324	EIF1AD
752	0.33	IQCH	-0.324	SSBP1
753	0.33	PDZD4	-0.324	HDAC2
754	0.33	ATMIN	-0.324	PEX14
755	0.33	CCDC53	-0.324	ARPC2

756	0.33	BECN1	-0.324	LOC100507373
757	0.33	GTF2I	-0.324	THBS1
758	0.33	SNX21	-0.324	HECA
759	0.33	GLP1R	-0.324	CYP2A7
760	0.33	DIABLO	-0.323	ATAD3B
761	0.33	PARP6	-0.323	TMEM209
762	0.33	OR2W1	-0.323	RPL27A
763	0.33	SDHA	-0.323	PDZD4
764	0.329	SF3B4	-0.323	TNRC6B
765	0.329	ITPKC	-0.323	ZNF337
766	0.329	CRTAP	-0.323	FAM24A
767	0.329	HCN2	-0.323	NME8
768	0.329	SFSWAP	-0.323	DLEU2
769	0.329	MAR7	-0.323	SMG1
770	0.329	ZNF501	-0.323	PRKCSH
771	0.329	UBE2B	-0.323	UBE2G2
772	0.329	EPHX2	-0.323	DEFA1
773	0.329	FAM110C	-0.323	GRIN2D
774	0.329	LRRC73	-0.323	FCRL2
775	0.328	DYNLRB2	-0.323	HAUS2
776	0.328	SLC11A2	-0.323	KCNA7
777	0.328	KLK2	-0.323	PABPC1
778	0.328	TNKS	-0.322	PXDNL
779	0.328	TRMT1L	-0.322	PTCH2
780	0.328	RNFT2	-0.322	FAM43A
781	0.328	NAP1L2	-0.322	ZNF75A
782	0.328	CDC14A	-0.322	A1BG-AS1
783	0.328	TOR3A	-0.322	MAGI1
784	0.328	ABCC3	-0.322	RNF182
785	0.328	SPZ1	-0.322	CHORDC1
786	0.328	ACER3	-0.322	LOC100506088
787	0.328	PTPRN	-0.322	CHCHD7
788	0.328	SLC7A11	-0.321	FKBP6
789	0.327	PLCD4	-0.321	KCNJ5
790	0.327	STRADA	-0.321	PIGA
791	0.327	CIAPIN1	-0.321	KRT75
792	0.327	RBM24	-0.321	CPSF1
793	0.327	ARL3	-0.321	POLRMT
794	0.327	PTRHD1	-0.321	C17orf104
795	0.327	PSMD7	-0.321	PKNOX1

796	0.327	CHD1L	-0.321	MIS18BP1
797	0.327	AKTIP	-0.321	GLYCTK
798	0.327	ERMAP	-0.321	ELOF1
799	0.327	ATP6V1E2	-0.32	EXTL3
800	0.327	PRKAA1	-0.32	RBM44
801	0.327	PDIA6	-0.32	PRDM2
802	0.327	PIP4K2C	-0.32	TRIM4
803	0.326	ANKRD54	-0.32	ACRBP
804	0.326	AHCYL1	-0.32	MICAL3
805	0.326	HAUS3	-0.32	PLEK
806	0.326	ACACA	-0.32	PPP6R1
807			-0.32	PPP4R1
808			-0.32	FUOM
809			-0.32	ACTR3
810			-0.319	ENAM
811			-0.319	NUDT4
812			-0.319	C2CD4A
813			-0.319	EXTL2
814			-0.319	SMIM1
815			-0.319	ETV6
816			-0.319	HTR4
817			-0.319	PPP1R12C
818			-0.319	GLMN
819			-0.319	LSM4
820			-0.319	KLF4
821			-0.319	FCRL4
822			-0.318	TCP11
823			-0.318	UBA52
824			-0.318	EEF2
825			-0.318	SP8
826			-0.318	SLC41A3
827			-0.318	L3MBTL2
828			-0.318	FAM209A
829			-0.318	PPIH
830			-0.318	MRPL37
831			-0.318	MROH1
832			-0.318	MLEC
833			-0.318	PTPN4
834			-0.318	C1QA
835			-0.318	LOC90499

836			-0.318	ATG5
837			-0.318	CCDC61
838			-0.318	AQPEP
839			-0.318	REXO4
840			-0.318	LILRB2
841			-0.317	GPR171
842			-0.317	C16orf95
843			-0.317	HSPA6
844			-0.317	HMGCLL1
845			-0.317	FAM194A
846			-0.317	FLJ43663
847			-0.317	VPS13A-AS1
848			-0.317	STX16
849			-0.317	DGAT1

Table S5. NCI-60/COMPARE analysis: summary of SLC genes whose mRNA expression is positively or negatively correlated with chemosensitivity to compound **1** ($\log GI_{50}$)

Gene	Pearson's R^{a}	Function	*P value*[b]
SLC11A2	0.328	H^{+}-coupled Cu and M^{2+} symporter	*
SLC12A4	0.352	K^{+}/Cl^{-} coupled transporter	**
SLC16A1	0.327	lactate/pyruvate transporter	*
SLC16A4	0.358	monocarboxylic acid transporter	**
SLC17A9	-0.366	ATP/mononucleotide vesicular uptake	**
SLC22A17	0.374	multi-specific cation transporter (brain)	**
SLC22A5	0.38	carnitine transporter	**
SLC22A7	-0.397	multi-specific anion transporter	**
SLC24A2	-0.427	Ca^{2+}/cation antiporter	***
SLC25A32	-0.318	mitochondrial folate transporter	*
SLC26A8	-0.36	anion transporter	**
SLC27A7	-0.352	fatty acid transporter	**
SLC2A9	-0.374	uric acid transporter GLUT9	**
SLC30A1	0.371	cation transporter	**
SLC30A5	0.404	Zn^{2+} transporter	**
SLC35E3	0.419	putative transporter	**
SLC35F6	0.353	carbohydrate/H^{+} symporter	**
SLC38A7	0.362	Na^{+} coupled amino acid transporter	**
SLC39A8	0.381	Zn^{2+} and Cd^{2+} transporter	**
SLC3A2	0.392	amino acid transporter	**
SLC41A3	-0.318	cation transporter	*
SLC43A3	-0.415	putative transporter	**
SLC47A1	**0.692**	**H^{+}-coupled organic cation antiporter**	*****
SLC48A1	0.38	heme transporter	**
SLC51B	0.393	bile acid transporter	**
SLC6A19	-0.38	Na^{+} dependent neutral amino acid transporter	**
SLC6A2	0.371	Na^{+}/neurotransmitter symporter	**
SLC7A11	0.328	anionic amino acid transporter	*
SLC8A1	0.364	Na^{+}/Ca^{2+} exchanger	**
SLCO4A1	-0.328	organic anion transporter	*

[a] $N = 58$. [b] * $P < 0.05$; ** $P < 0.01$; *** $P < 0.001$; ***** $P < 0.00001$.

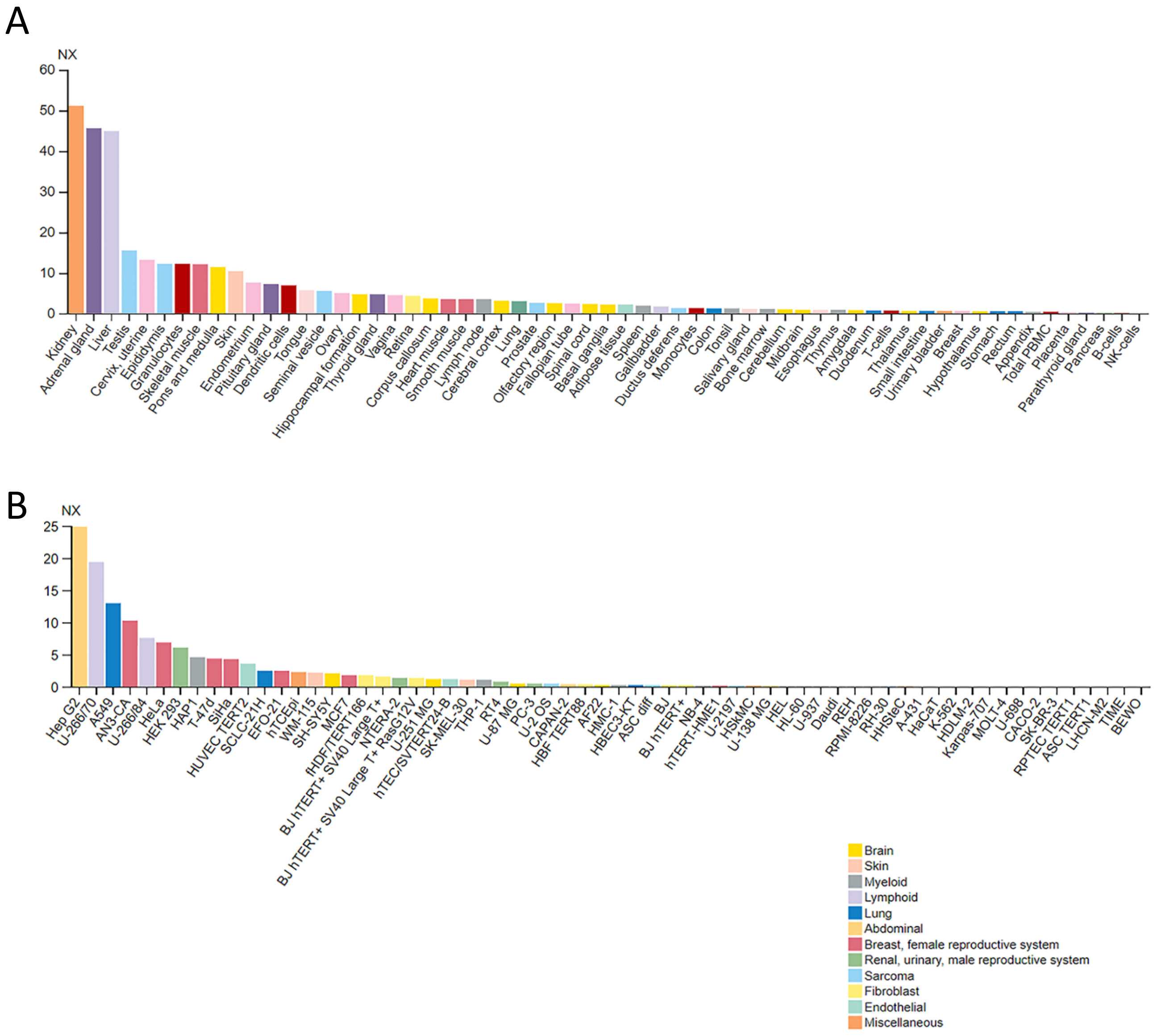

Figure S2. Expression of hMATE1 (*SLC47A1*) in normal human tissue (A) and in cancer cells (B); The Human Protein Atlas, version 19.1, 12/2019, www.proteinatlas.org.

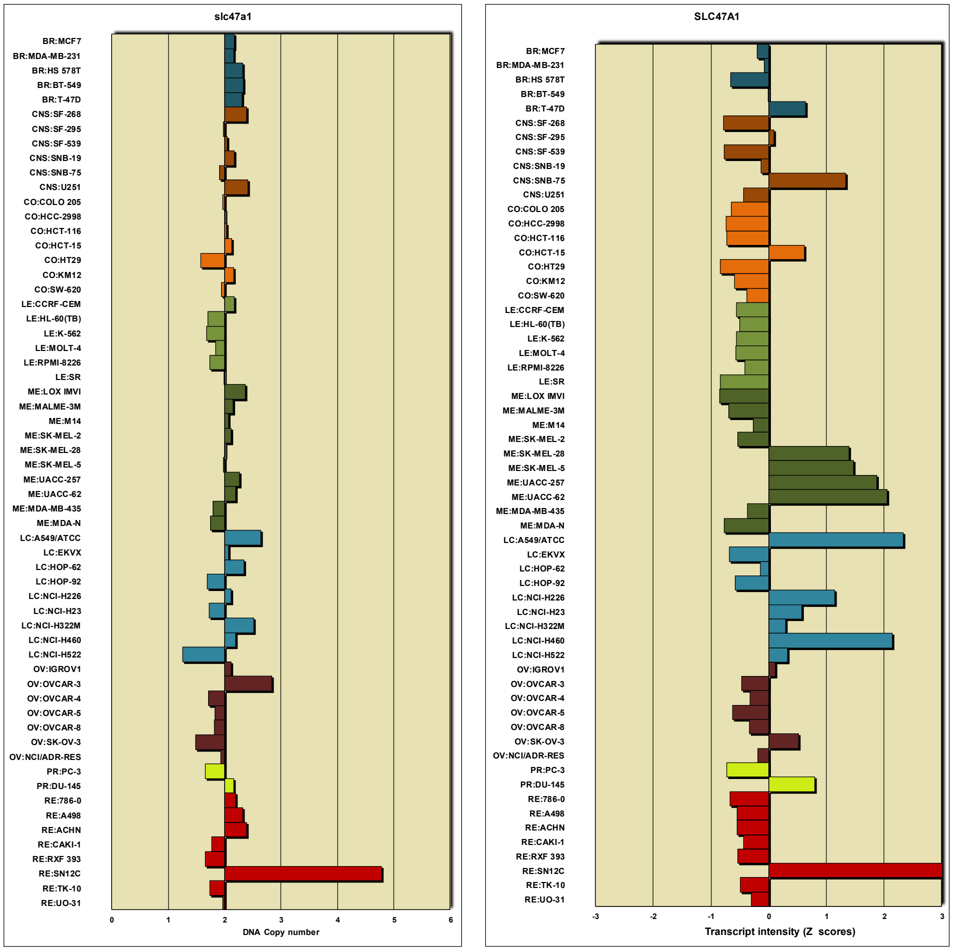

Figure S3. *SLC47A1* gene copy numbers (left) and transcript levels (right) in NCI-60 (NCI CellMiner analysis tool, database version 2.2)

Table S6. Summary of top 10 overlaps[a] between gene ontology (GO) gene sets of the Molecular Signatures Database (MSigDB)[b] and the input gene set[c] derived from NCI-60 COMPARE correlation between activity of compound **1** (GI_{50}) and gene transcript levels for *SLC47A1*

	**MSigDB Gene Set**	***K*[d]**	**Description**	***k*[e]**	***k/K***	***p*-value[f]**	**FDR *q*-value**
**1**	GO_INTRACELLULAR_TRANSPORT	1825	The directed movement of substances within a cell. [GOC:ai]	101	0.056	2.07 e^{-19}	2.07 e^{-15}
**2**	GO_CELLULAR_MACROMOLECULE_LOCALIZATION	1897	Any process in which a macromolecule is transported to, and/or maintained in, a specific location at the level of a cell. Localization at the cellular level encompasses movement within the cell, from within the cell to the cell surface, or from one location to another at the surface of a cell. [GOC:mah]	102	0.054	1.01 e^{-18}	5.02 e^{-15}
**3**	GO_CELL_PROJECTION_PART	1440	Any constituent part of a cell projection, a prolongation or process extending from a cell, e.g. a flagellum or axon. [GOC:jl]	85	0.059	4.35 e^{-18}	1.45 e^{-14}
**4**	GO_WHOLE_MEMBRANE	1653	Any lipid bilayer that completely encloses some structure, and all the proteins embedded in it or attached to it. Examples include the plasma membrane and most organelle membranes. [GOC:dos]	91	0.055	2.14 e^{-17}	5.34 e^{-14}
**5**	GO_INTRACELLULAR_PROTEIN_TRANSPORT	1164	The directed movement of proteins in a cell, including the movement of proteins between specific compartments or structures within a cell, such as organelles of a eukaryotic cell. [GOC:mah]	73	0.063	4.97 e^{-17}	9.93 e^{-14}
**6**	GO_PROTEOLYSIS	1762	The hydrolysis of proteins into smaller polypeptides and/or amino acids by cleavage of their peptide bonds. [GOC:bf, GOC:mah]	93	0.053	1.3 e^{-16}	2.16 e^{-13}

**7**	GO_NEURON_PART	1715	Any constituent part of a neuron, the basic cellular unit of nervous tissue. A typical neuron consists of a cell body (often called the soma), an axon, and dendrites. Their purpose is to receive, conduct, and transmit impulses in the nervous system. [GOC:pr, http://en.wikipedia.org/wiki/Neuron]	91	0.053	$2.07\ e^{-16}$	$2.95\ e^{-13}$
**8**	GO_CELL_PROJECTION_ORGANIZATION	1512	A process that is carried out at the cellular level which results in the assembly, arrangement of constituent parts, or disassembly of a prolongation or process extending from a cell, e.g. a flagellum or axon. [GOC:jl, GOC:mah, http://www.cogsci.princeton.edu/~wn/]	84	0.056	$2.43\ e^{-16}$	$3.04\ e^{-13}$
**9**	GO_RIBONUCLEOTIDE_BINDING	1891	Interacting selectively and non-covalently with a ribonucleotide, any compound consisting of a ribonucleoside that is esterified with (ortho)phosphate or an oligophosphate at any hydroxyl group on the ribose moiety. [GOC:mah]	95	0.050	$1.29\ e^{-15}$	$1.43\ e^{-12}$
**10**	GO_MICROTUBULE_BASED_PROCESS	734	Any cellular process that depends upon or alters the microtubule cytoskeleton, that part of the cytoskeleton comprising microtubules and their associated proteins. [GOC:mah]	53	0.072	$4.34\ e^{-15}$	$4.34\ e^{-12}$

[a] Number of genes in comparison (*n*): 785, number of genes in universe (*N*): 38055. [b] MSigDB database v6.2, updated July 2018; GSEA/MSigDB website v6.3, released 01/2018 by the Broad Institute Inc. [c] See Table S4. Number of positively correlated genes: 806, $P < 0.05$. [d] Number of genes in gene set. [e] Number of genes in overlap. [f] For hypergeometric distribution.

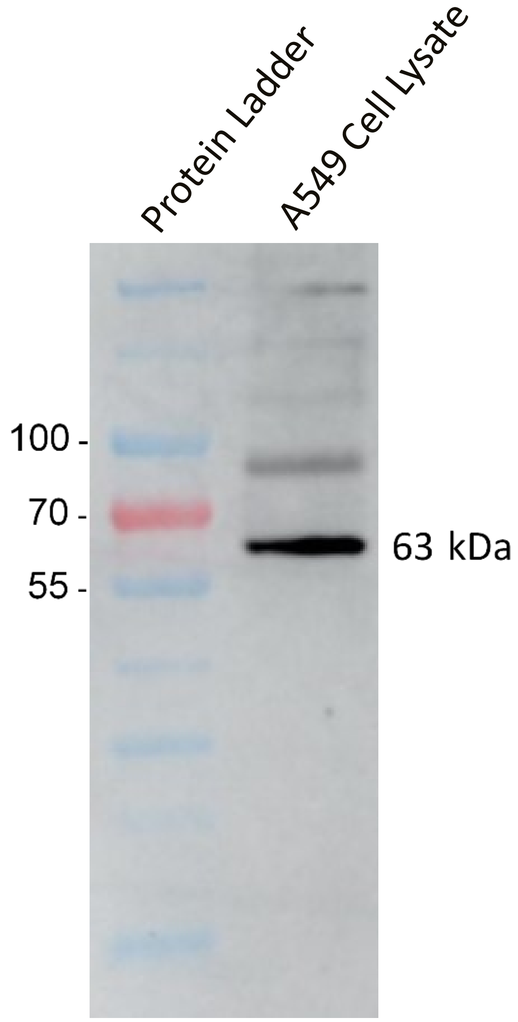

Figure S4. Western blot analysis of A549 cell lysate for hMATE1 expression. The blot shows a band consistent with the 63-kDa full-length, 586-amino acid protein (The Human Protein Atlas, version 19.1, 12/2019, www.proteinatlas.org).

A

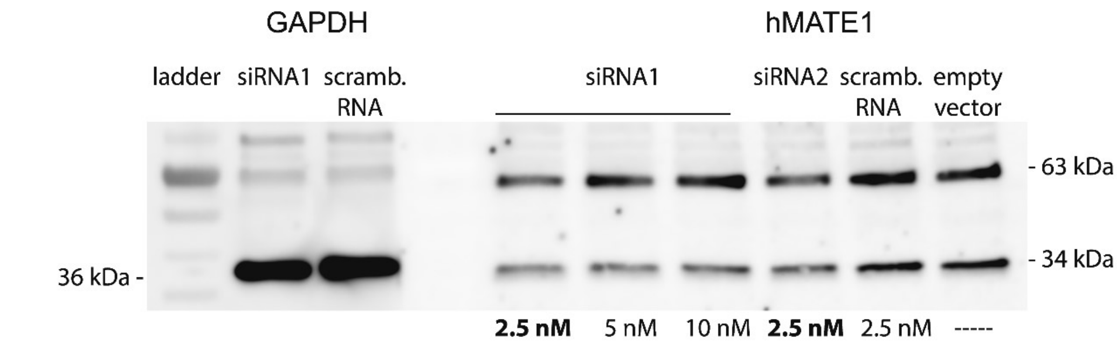

B

```
34 kDa: 10         20         30         40         50
MCPGLPRPMS RSSFQLFLQP FFICYKLNIC STRLSSSPMA RSPGNVCVPP
        60         70         80         90        100
TGWSLECLQD WASFLRLAIP SMLMLCMEWW AYEVGSFLSG ILGMVELGAQ
       110        120        130        140        150
SIVYELAIIV YMVPAGFSVA ASVRVGNALG AGDMEQARKS STVSLLITVL
       160        170        180        190        200
FAVAFSVLLL SCKDHVGYIF TTDRDIINLV AQVVPIYAVS HLFEALACTS
       210        220        230        240        250
GGVLRGSGNQ KVGAIVNTIG YYVVGLPIGI ALMFATTLGV MGCPENLEGI
       260        270        280        290        300
LTNDVGKTGE PQSDQQMRQE EPLPEHPQDG AKLSRKQLVL RRGLLLLGVF
       310
LILLVGILVR FYVRIQ
```

```
63k Da: 10         20         30         40         50
MEAPEEPAPV RGGPEATLEV RGSRCLRLSA FREELRALLV LAGPAFLVQL
        60         70         80         90        100
MVFLISFISS VFCGHLGKLE LDAVTLAIAV INVTGVSVGF GLSSACDTLI
       110        120        130        140        150
SQTYGSQNLK HVGVILQRSA LVLLLCCFPC WALFLNTQHI LLLFRQDPDV
       160        170        180        190        200
SRLTQTYVTI FIPALPATFL YMLQVKYLLN QGIVLPQIVT GVAANLVNAL
       210        220        230        240        250
ANYLFLHQLH LGVIGSALAN LISQYTLALL LFLYILGKKL HQATWGGWSL
       260        270        280        290        300
ECLQDWASFL RLAIPSMLML CMEWWAYEVG SFLSGILGMV ELGAQSIVYE
       310        320        330        340        350
LAIIVYMVPA GFSVAASVRV GNALGAGDME QARKSSTVSL LITVLFAVAF
       360        370        380        390        400
SVLLLSCKDH VGYIFTTDRD IINLVAQVVP IYAVSHLFEA LACTSGGVLR
       410        420        430        440        450
GSGNQKVGAI VNTIGYYVVG LPIGIALMFA TTLGVMGLWS GIIICTVFQA
       460        470        480        490        500
VCFLGFIIQL NWKKACQQAQ VHANLKVNNV PRSGNSALPQ DPLHPGCPEN
       510        520        530        540        550
LEGILTNDVG KTGEPQSDQQ MRQEEPLPEH PQDGAKLSRK QLVLRRGLLL
       560        570
LGVFLILLVG ILVRFYVRIQ
```

C D

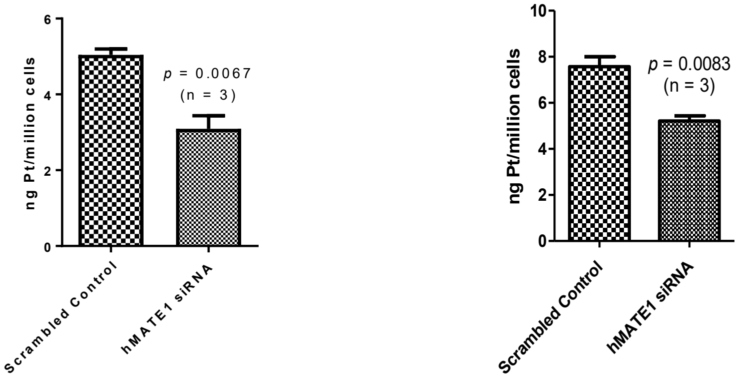

Figure S5. (A) Western blot for optimized RNAi conditions. The full-length protein (63 kDa) and the 34-kDa splice variant are observed. Transfection with 2.5 nM of both siRNA1 and siRNA2 provided the best knockdown efficiency relative to scrambled control and empty vector (lipofectamine). The GAPDH loading control is also shown. The gel image (30 sec. exposure) was contrast enhanced but otherwise not altered. (B) Rabbit–anti-human hMATE1 antibodies used in this study and aligned amino-acid sequences of the 63-kDa full-length protein and the 34-kDa splice variant (NCBI). Specific antibodies/epitopes: underlined: Thermo Fisher PA5-25272 and AVIVA OAAB02770 (residues 492-519, underlined), based on full length protein); bold: Abcam, ab104016 (**residues 500-530**, **bold**, based on full length protein). (C,D) Results for two additional drug uptake experiments after hMATE1 (*SLC47A1*) knockdown, quantified by ICP-MS, showing reduction in uptake of compound **1** by 39% ($p < 0.01$) and 32% ($p < 0.01$), respectively.

Table S7. Pattern comparisons for *SLC47A1* expression in NCI CellMiner.

**CellMiner Address:**	https://discover.nci.nih.gov/cellminer/
**05-14-2019**	Pattern Comparison
**CellMiner Database Version:**	2.2
**Human Genome Version:**	HG 19
**Date:**	04-17-2019
**Input data:**	**SLC47A1**
**Number of genes:**	25683
**Number of drugs:**	21738
**Number of miRNA:**	361

Deposited as Excel spreadsheet

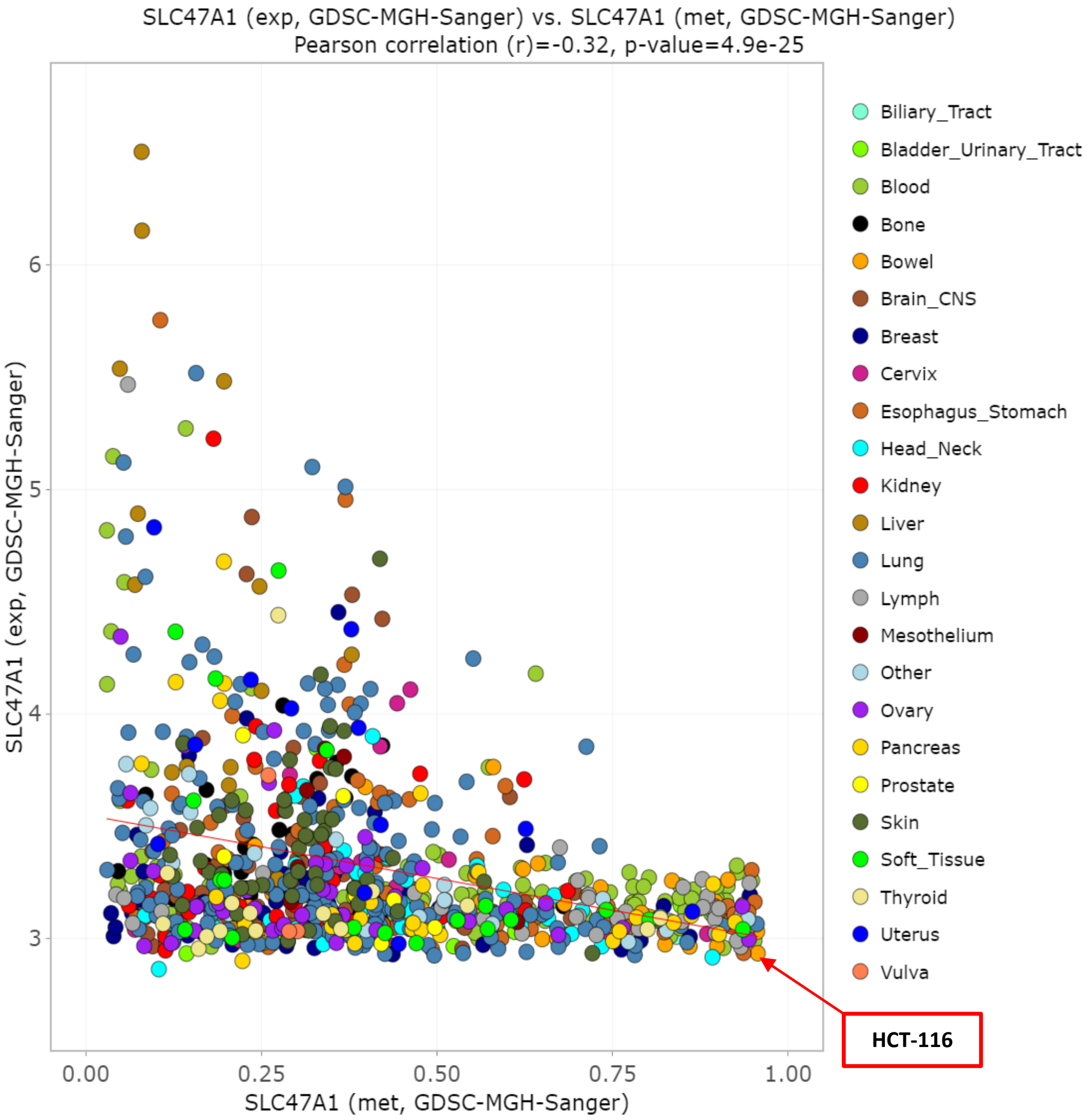

Figure S6. A highly significant correlation ($R = -0.32$, $p = 4.9 \times 10^{-25}$) between CPI methylation status and expression levels of the *SLC47A1* gene is observed in 963 cancer cell lines of the Genomics of Drug Sensitivity in Cancer (GDSC) database (CellMinerCDB, version 1.1, discover.nci.nih.gov/cellminercdb). The data point for the colorectal cancer cell line HCT-116 used in this study is highlighted.

Table S8. Summary of significant ($p < 0.05$) correlations identified between CGI methylation status and expression levels of the *SLC47A1* gene in 963 cancer cell lines[a] of different tissues of origin and cell types

**Cell Line Origin**	**Pearson's *R***	***P* value**
Multiple Myeloma	–0.55	0.028
Colon	–0.33	0.025
Esophagus/Stomach	–0.41	7.6×10^{-4}
Liver	–0.56	0.02
**Lung**	**–0.32**	$\mathbf{8.2 \times 10^{-6}}$
**NSCLC**	**–0.43**	$\mathbf{1.1 \times 10^{-5}}$
**Lung Adenocarcinoma**	**–0.47**	$\mathbf{4.7 \times 10^{-4}}$
Ovaries	–0.32	0.032
Pancreas	–0.47	8.1×10^{-3}
Epithelial	–0.16	4.4×10^{-3}
Epithelial–Mesenchymal	–0.43	2.7×10^{-7}
Mesenchymal	–0.23	1.3×10^{-5}

[a] Genomics of Drug Sensitivity in Cancer (GDSC) database (CellMinerCDB, version 1.1, discover.nci.nih.gov/cellminercdb).

Table S9. Summary of correlations observed for chemosensitivity and omics data for compound **1**.

**Data in Comparison**		**Pearson's *R***	***P* value**
NCI-60, $logGI_{50}$	*SLC47A1* transcript level	0.692[a]	< .00001 (*****)[a]
NCI-60, $logGI_{50}$	*DNMT1* transcript level	-0.378[a]	0.0034 (**)[a]
SLC47A1 transcript level	*SLC47A1* CGI methylation	-0.416[b]	< 0.001 (***)[b]
SLC47A1 transcript level	*SLC47A1* gene copy number	0.398[b]	< 0.001 (***)[b]
SLC47A1 transcript level	*EZH2* transcript level	-0.289[b]	0.025 (*)[b]
SLC47A1 CGI methylation	*DNMT1* transcript level	0.311[b]	0.015 (*)[b]
EZH2 transcript level	*DNMT1* transcript level	0.479[b]	< 0.001 (***)[b]

[a] NCI COMPARE analysis, *n* = 58. [b] NCI CellMiner analysis tool, database version 2.2, *n* = 60.

Table S10. Summary of top 10 overlaps[a] between all gene sets of the molecular signatures database (MSigDB)[b] and the input gene set[c] of hypermethylated genes negatively correlated with *SLC47A1* expression.

	MSigDB Gene Set	*K*[d]	Description	*k*[e]	*k/K*	*p*-value[f]	FDR *q*-value
**1**	BENPORATH_ES_WITH_H3K27ME3	1118	Set 'H3K27 bound': genes possessing the trimethylated H3K27 (H3K27me3) mark in their promoters in human embryonic stem cells, as identified by ChIP on chip.	115	0.1029	1.21 e^{-92}	2.15 e^{-88}
**2**	BENPORATH_EED_TARGETS	1062	Set 'Eed targets': genes identified by ChIP on chip as targets of the Polycomb protein EED [GeneID=8726] in human embryonic stem cells.	111	0.1045	5.70 e^{-90}	5.08 e^{-86}
**3**	MIKKELSEN_MEF_HCP_WITH_H3K27ME3	590	Genes with high-CpG-density promoters (HCP) bearing histone H3 trimethylation mark at K27 (H3K27me3) in MEF cells (embryonic fibroblast).	91	0.1542	1.23 e^{-88}	7.28 e^{-85}
**4**	BENPORATH_SUZ12_TARGETS	1038	Set 'Suz12 targets': genes identified by ChIP on chip as targets of the Polycomb protein SUZ12 [GeneID=23512] in human embryonic stem cells.	108	0.104	3.62 e^{-87}	1.61 e^{-83}
**5**	BENPORATH_PRC2_TARGETS	652	Set 'PRC2 targets': Polycomb Repression Complex 2 (PRC) targets; identified by ChIP on chip on human embryonic stem cells as genes that: posess the trimethylated H3K27 mark in their promoters and are bound by SUZ12 [GeneID=23512] and EED [GeneID=8726] Polycomb proteins.	75	0.115	7.73 e^{-63}	2.75 e^{-59}
**6**	MEISSNER_BRAIN_HCP_WITH_H3K4ME3_AND_H3K27ME3	1069	Genes with high-CpG-density promoters (HCP) bearing histone H3 dimethylation at K4 (H3K4me2) and trimethylation at K27 (H3K27me3) in brain.	89	0.0833	1.21 e^{-62}	3.60 e^{-59}

**7**	GO_INTRINSIC_COMPONENT_OF_PLASMA_MEMBRANE	1649	The component of the plasma membrane consisting of the gene products and protein complexes having either part of their peptide sequence embedded in the hydrophobic region of the membrane or some other covalently attached group such as a GPI anchor that is similarly embedded in the membrane.	104	0.0631	7.53 e^{-62}	1.92 e^{-58}
**8**	MEISSNER_NPC_HCP_WITH_H3K4ME2_AND_H3K27ME3	349	Genes with high-CpG-density promoters (HCP) bearing histone H3 dimethylation mark at K4 (H3K4me2) and trimethylation mark at K27 (H3K27me3) in neural precursor cells (NPC).	60	0.1719	7.26 e^{-61}	1.62 e^{-57}
**9**	MIKKELSEN_NPC_HCP_WITH_H3K27ME3	341	Genes with high-CpG-density promoters (HCP) bearing histone H3 trimethylation mark at K27 (H3K27me3) in neural progenitor cells (NPC).	58	0.1701	1.54 e^{-58}	3.04 e^{-55}
**10**	MIKKELSEN_MCV6_HCP_WITH_H3K27ME3	435	Genes with high-CpG-density promoters (HCP) bearing the tri-methylation mark at H3K27 (H3K27me3) in MCV6 cells (embryonic fibroblasts trapped in a differentiated state).	60	0.1379	6.82 e^{-55}	1.21 e^{-51}

[a] Number of genes in comparison (*n*): 402, number of genes in universe (*N*): 38055. [b] MSigDB database v6.2, updated July 2018; GSEA/MSigDB website v6.3, released 01/2018 by the Broad Institute Inc. [c] See Table S7. Number of negatively correlated genes: 452, $P < 0.05$. [d] Number of genes in gene set. [e] Number of genes in overlap. [f] For hypergeometric distribution.

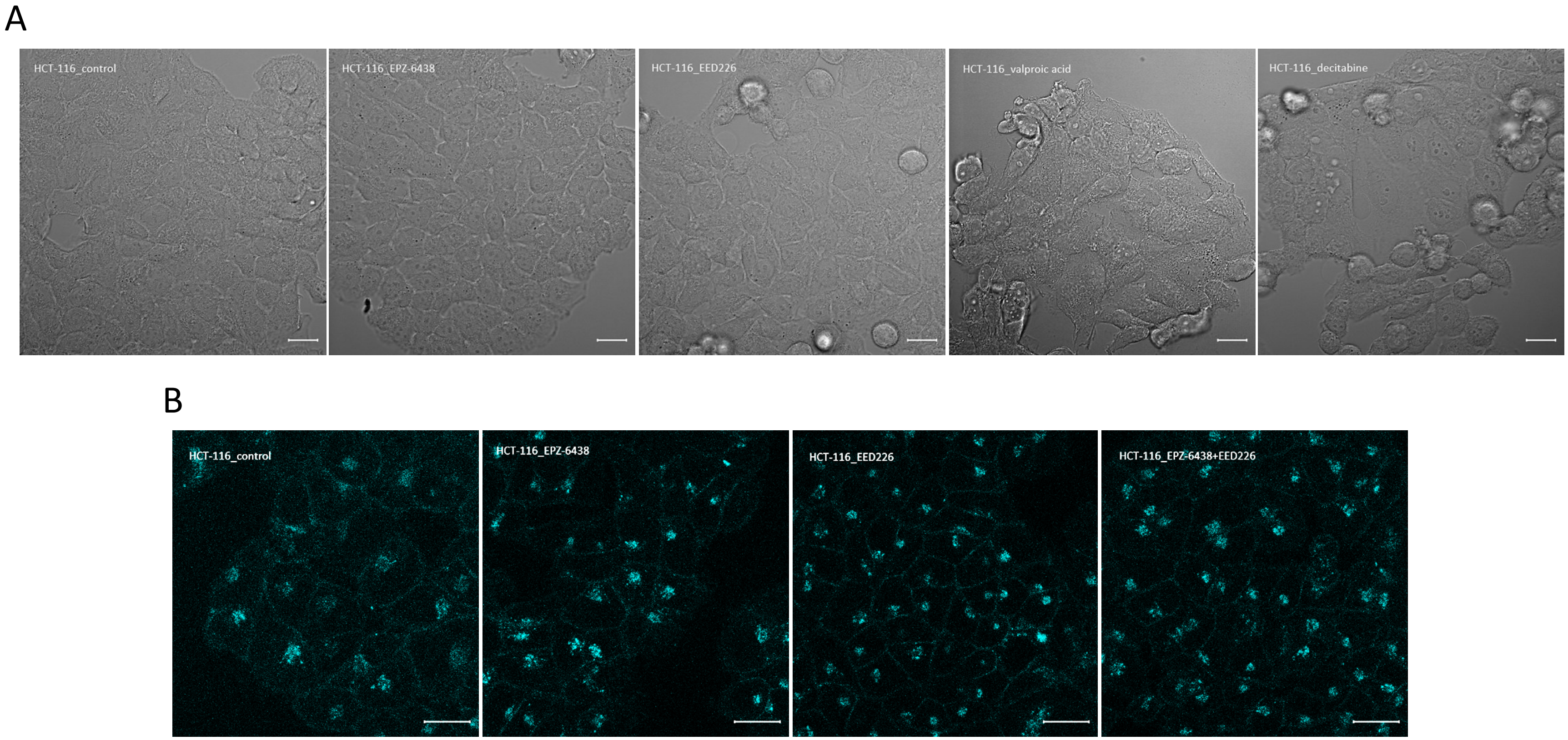

Figure S7. Pre-screening of HCT-116 colon cancer cells for the effects of epigenetic drugs on cell viability and accumulation of compound **1**. Cells were treated with single drugs or combinations of up to four epigenetic drugs at 2.5 µM for 72 hours. Cells were then fixed and imaged in the bright-field and blue fluorescence channels. (A) Bright-field images show intact monolayers of viable cells after incubation with EPZ-6438 and EED226 similar to DMSO-treated cells (control) but show changes in morphology and cell death after treatment with valproic acid and decitabine (only selected images of single treatments are shown). (B) Treatment at 2.5 µM EPZ-6438 and EED226, or a combination of the two drugs, for 72 hours leads to enhanced cellular accumulation of compound **1**, based on increased acridine-associated blue fluorescence localized to regions previously identified as vesicular structures (see the main text). Scale bars in (A) and (B) are 20 µm.

A

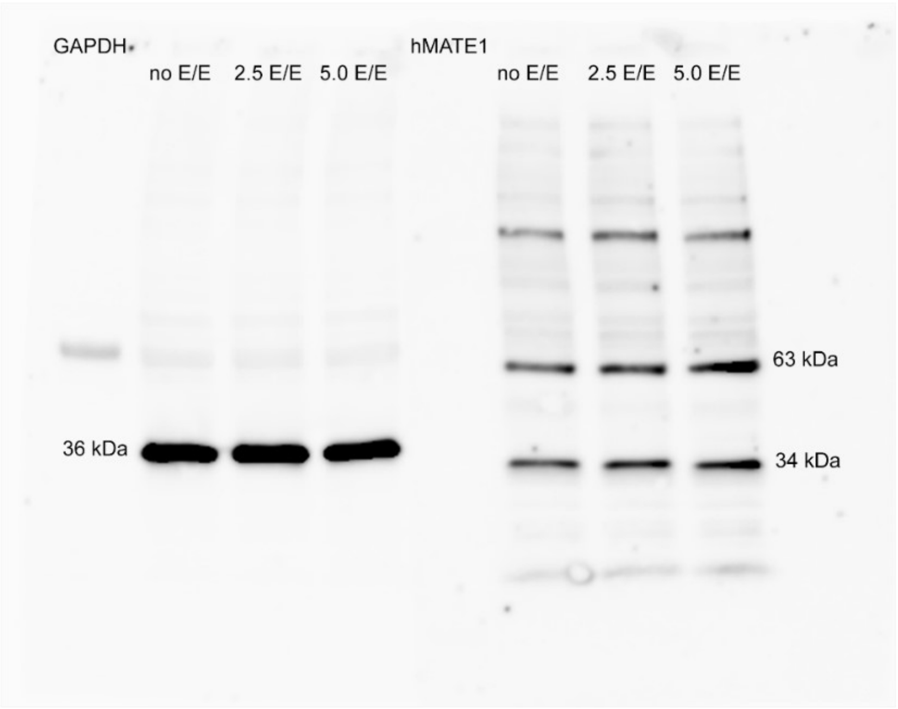

B

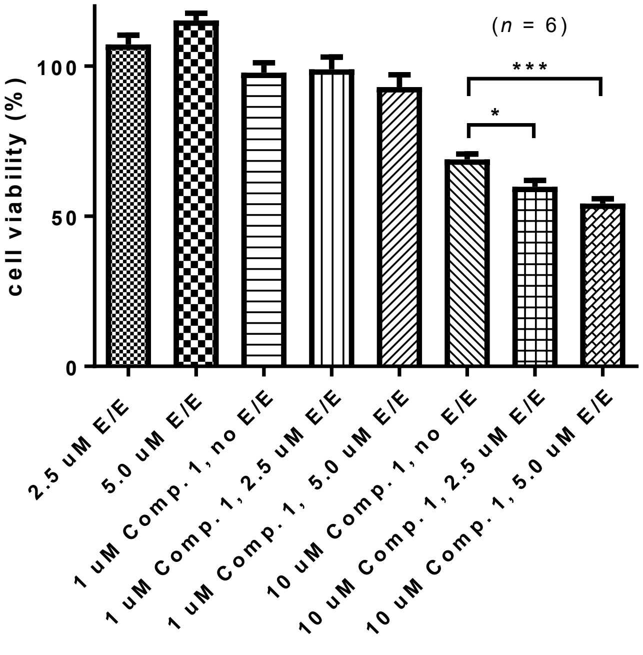

Figure S8. (A) Western blot analysis of hMATE1 protein in HCT-116 cell lysates pre-treated with a combination of EPZ-6438 and EED226 (E/E). Bands for GAPDH (36 kDa), full-length hMATE1 (63 kDa), and a splice variant of hMATE1 containing the antibody epitope (34 kDa) are labeled. (B) Cell proliferation assay for HCT-116 cells pre-exposed to 2.5 µM or 5 µM E/E for 72 hours and subsequently treated with 1 µM or 10 µM compound **1** for another 72 hours. A pronounced decrease in cell viability was observed at higher doses of epigenetic drugs. The data is presented as the mean ± S.E.M. for an assay performed in sextuplicate (*, $p < 0.05$; ***, $p < 0.001$; one-way ANOVA with post hoc test).

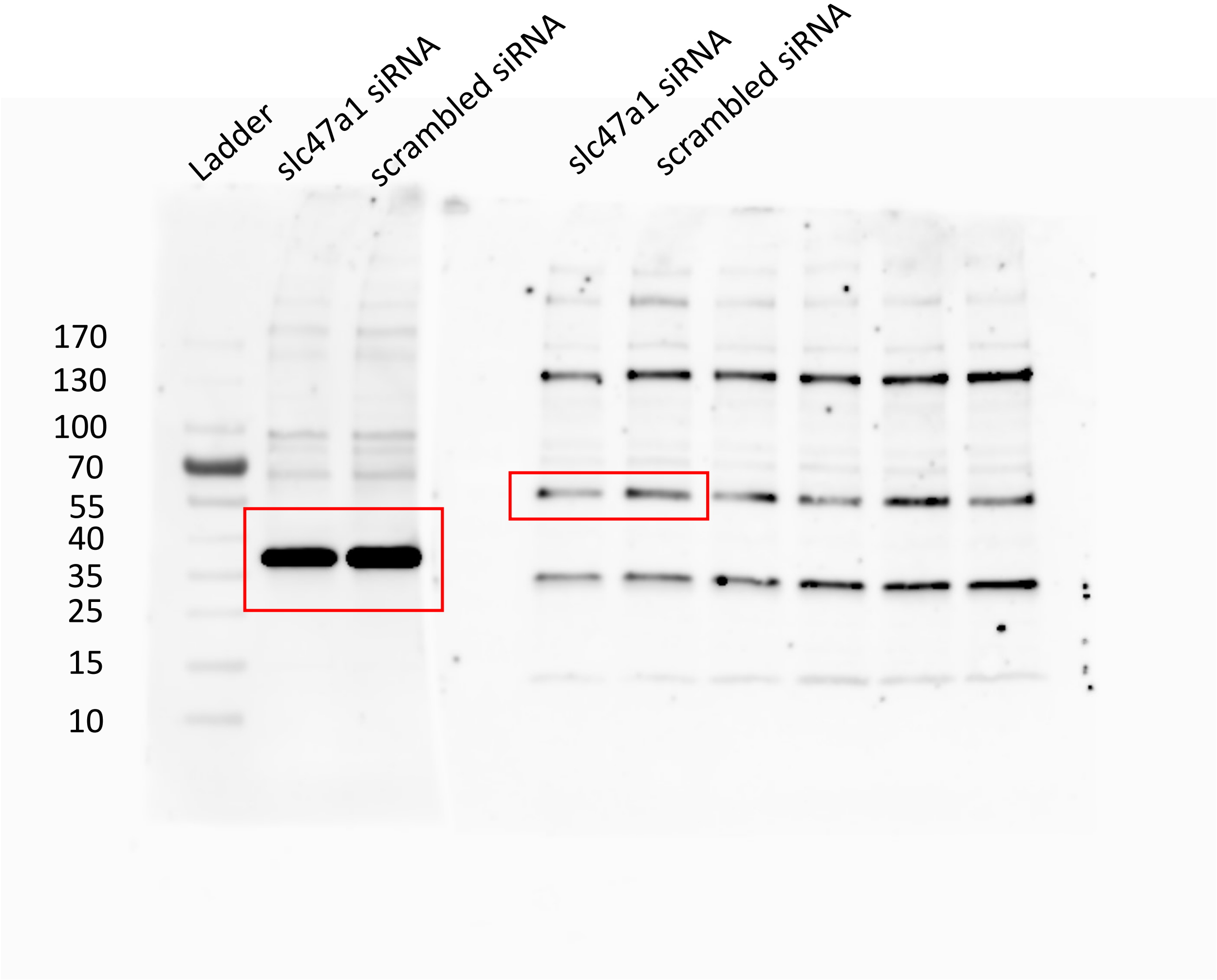

Figure S9. Full-length, uncropped/unprocessed image of Western blots presented in Fig. 3a in the main text for GAPDH protein (control, left) and hMATE1 protein (right). Exposure time: 3 min. Cropped sections used in Fig. 3a are highlighted with red boxes. To maintain a consistent presentation throughout the paper, the blot images in Fig. 3a have been flipped horizontally.

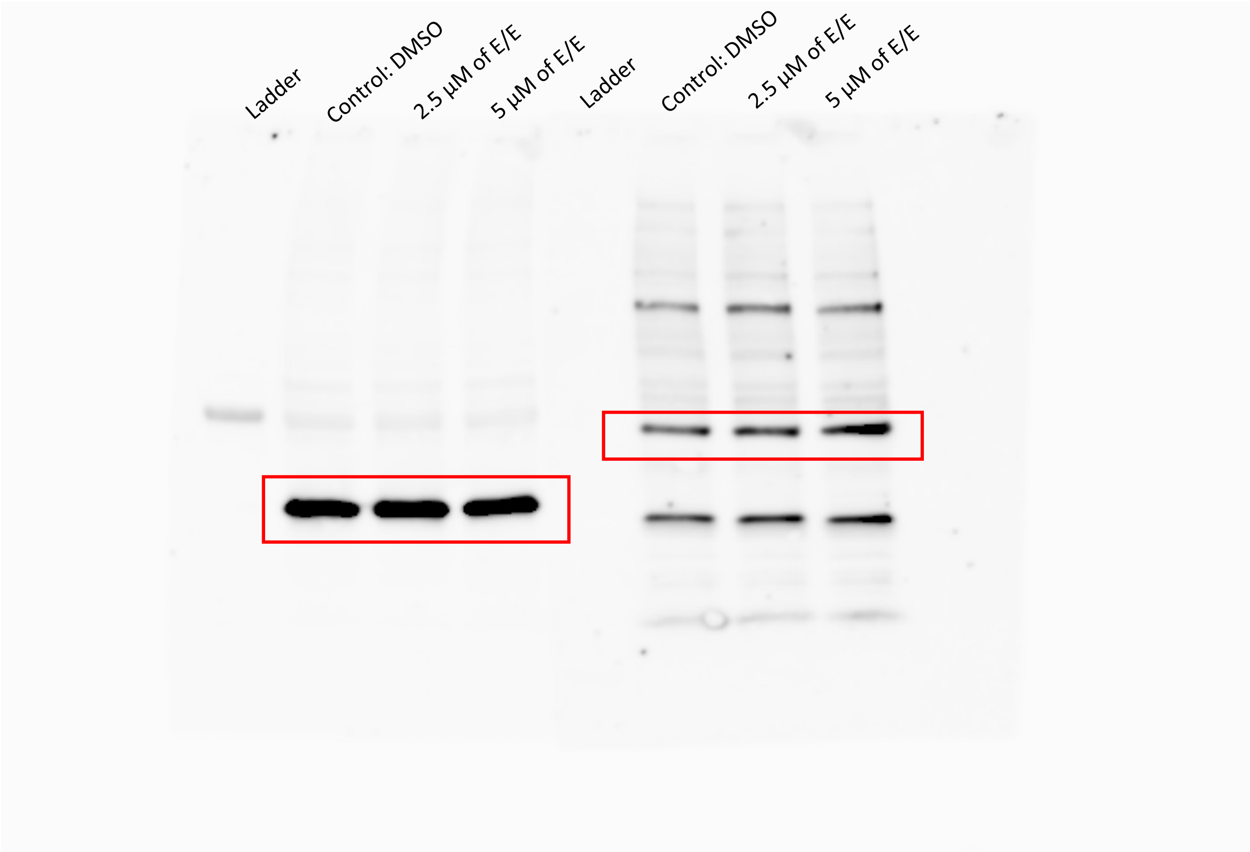

Figure S10. Full-length, uncropped/unprocessed image of Western blots presented in Fig. 4f in the main text for GAPDH protein (control, left) and hMATE1 protein (right). Exposure time: 20 sec. Cropped sections used in Fig. 4f are highlighted with red boxes.

Assay Layouts

AL1. Uptake of compound **1** into HCT-116 cells after sensitization with epigenetic drugs[a] monitored by confocal fluorescence microscopy (pre-screening).

Control	VA	VA EPZ	VA	VA EED EPZ	VAL DEC EPZ
Control	VA	EPZ	EPZ VA	EPZ EED DEC	EPZ DEC VAL EED
Control	EED	EED EPZ	EED VA	EED	EED DEC EPZ
Control	DEC	DEC EPZ	DEC VA	DEC EED	DEC

[a] VA, valproic acid; DEC, decitabine; EPZ, EPZ-6438; EED, EED226.

AL2. Uptake of compound **1** into HCT-116 cells after sensitization with EPZ-6438, EED226, and a combination of both drugs at escalating doses monitored by confocal fluorescence microscopy.

Control	2.5 µM	5 µM	10 µM	20 µM	50 µM
Control	2.5 µM	5 µM	10 µM	20 µM	50 µM
Control	2.5 µM	5 µM	10 µM	20 µM	50 µM

Green: EPZ-6438, Blue: EED226, Red: EPZ-6438 + EED226 (“E/E”), 1:1

AL3. Cell proliferation assay for A549 cells after hMATE1 (*SLC47A1*) knockdown.

	1	2	3	4	5	6	7
A	PBS	PBS	PBS	PBS	PBS	PBS	PBS
B	PBS	Comp. 1		Comp. 1		blank	PBS
C	PBS						PBS
D	PBS						PBS
E	PBS	PBS	PBS	PBS	PBS	PBS	PBS

Assembly: Add 20 µL Opti-Mem into wells B2-5, C2-6, and D2-5. Add 23.2 µL of Opti-Mem into wells B6, C6, D6. Then add siRNA to B2-3, C2-3, and D2-3. Add scrambled RNA to B4-5, C4-5, and D4-5. Add diluted lipofectamine (siRNA:lipo 6:1) to B2-5, C2-5, and D2-5. Transfer cells to B2-5, C2-5, and D2-5 at a density of 5000 cells/well. Add same amount of medium into well B6-D6 as blank control. Add PBS into the outermost wells to prevent evaporation of transfection reactions. B2, C2, and D2 are siRNA

+ compound **1**; B3, C3, and D3 are siRNA only; B4, C4, and D4 are scrambled RNA + compound **1**; B5, C5, and D5 are scrambled RNA only; B6, C6, and D6 are blanks.

AL4. Cell proliferation assay for HCT-116 cells after exposure to epigenetic drugs.

	1	2	3	4	5	6	7	8	9	10	11
A	PBS	PBS	PBS	PBS	PBS	PBS	PBS	PBS	PBS	PBS	PBS
B	PBS	No drug	1 µM	10 µM	No drug	No drug	1 µM	1 µM	10 µM	10 µM	PBS
C	PBS										PBS
D	PBS	⬇	⬇	⬇	⬇	⬇	⬇	⬇	⬇	⬇	PBS
E	PBS										PBS
F	PBS										PBS
G	PBS										PBS
H	PBS	PBS	PBS	PBS	PBS	PBS	PBS	PBS	PBS	PBS	PBS

Assembly: Add EED-226 and EPZ-6438 ("E/E") to the cells in wells B5-G5, B7-G7, and B9-G9 to produce a final concentration of 2.5 µM, and to wells B6-G6, B8-G8, and B10-G10 to a final concentration of 5 µM. Incubate for a total of 72 hours and replace medium with fresh epi-drug(s) every 24 hours. Replace epi-drug after 72 hours with compound **1** at a final concentration of 1 µM in wells B3-G3, B7-G7, and B8-G8, and 10 µM in wells B4-G4, B9-G9, and B10-G10. Yellow columns: 2.5 µM E/E; Blue columns: 5 µM E/E. Column 2 is a no-treatment control, columns 3 and 4 are no-sensitization controls.

Experimental Details for Assays

Assays and sample preparation for confocal microscopy analysis

For transporter inhibition assays, cells were seeded into 35-mm poly-D-lysine-coated glass bottom dishes (MatTek Corporation, Ashland, MA, USA) at a density of 10^5 cells/mL in 2 mL of medium per dish. Cells were allowed to attached overnight prior to pre-treatment with 10 μM pyrimethamine or vehicle for 20 minutes and subsequent treatment with 10 μM compound **1** for 4 hours. Medium was removed and dishes were washed 3 times with 1 mL of pre-warmed PBS buffer. Cells were then fixed with 4% formaldehyde in PBS (Thermo Fisher) for 15 minutes at room temperature and washed an additional 3 times with PBS before imaging. The fluorescence of acridine was excited with a 405 nm (15 mW) laser at 4.4% and collected between 405-481 nm. The intensity of acridine-related fluorescence in treated cells was estimated by drawing a region of interest (ROI) around each cell, with the bright-field images assisting in identifying the cell perimeter. Signal intensities are averages from all pixels in the ROI minus the background fluorescence. A total of more than 100 individual cells across 4 views of 2 independent experiments were analyzed in this fashion. Automated quantification of fluorescence intensities was also performed using CellProfiler 3.0[3] with similar results (data not shown).

For RNAi knockdown of hMATE1 (*SLC47A1*) in imaging assays, A549 cells were harvested from T-75 cell culture flasks and seeded on a 6-well plate at a density of 150,000 cells per well. Silencer Select siRNA1, Silencer Select siRNA2, and scrambled siRNA (Silencer Select Negative Control #1) were thoroughly mixed with RNAiMAX in Opti-Mem media according to manufacturer's protocol and added to each well. Cells transfected at a final siRNA concentration of 20 nM for 48 hours at 37 °C were detached with trypsin and subcultured at a 1:2 ratio into 35 mm MatTek plates and allowed to attach overnight. Cell culture medium was replaced and supplemented with 10 μM compound **1**, and dishes were incubated for 4 hours at 37 °C. Cells were fixed with 4% formaldehyde, permeabilized with 0.5% Triton X-100, washed with PBS, incubated with 7.5% BSA) for 30 minutes at room temperature, and incubated with appropriately diluted primary antibody in 1% BSA for 1 hour at room temperature. After the cells were washed with PBS, they were incubated with the secondary antibody (Goat-anti-Rabbit IgG Alexa Fluor-635), diluted in 1% BSA (1:400 anti-rabbit) for 1 hour at 37 °C. After three PBS washes, samples were imaged immediately or stored in PBS at 4 °C for further testing.

For sensitization assays, HCT-116 cells were harvested from T-75 flasks, seeded on a 24-well plate with glass-like polymer bottom (P24-1.5P, Cellvis, Sunnyvale, CA) with 25,000 cells per well, and allowed to attached overnight. Single drugs or drug combinations were tested in this assay at final concentrations of 2.5 μM EED226, 2.5 μM EPZ-6438, 500 μM valproic acid, and 10 μM decitabine (see

assay layout 1, **AL1**, in the SI). Cells were incubated at 37 °C for 72 hours. Each well was replaced with fresh medium supplemented with 10 μM compound **1**, and incubation was continued for 4 additional hours. Each well was washed with 3 times with warm PBS, before cells were fixed with 0.5 mL of 4% formaldehyde at room temperature for 15 minutes. After 3 PBS washes, plates were immediately imaged or stored at 4 °C until analyzed. Subsequent incubations of HCT-116 cells at escalating doses of EED226 and EPZ-6438 were performed analogously (see **AL2** in the SI). Representative conditions that were screened for expression levels of hMATE1 were determined by immunofluorescence with anti-hMATE1 antibody as described in RNAi knockdown experiments.

Assays and sample preparation for ICP-MS analysis

For transporter inhibition assays, 700,000 A549 cells in 2.5 mL of F12K media (ATCC 30-2004), supplemented with 10% FBS, and 10% penstrep, and 10% L-glutamine (Thermo Fisher, 25030-081), were seeded into T-25 flasks and allowed to attach overnight. Cells pre-treated with 100 nM pyrimethamine for 25 minutes and untreated cells were then dosed with 100 nM compound **1** for 3 hours. After treatment, medium was aspirated, and cells were washed 3 times with fresh media. Trypsin was added to detach cells, and 3 mL of fresh media were added to each flask to collect the cell suspensions, which were pelleted by centrifugation at 250 × g for 3 minutes. After the supernatant was aspirated, pellets were washed with 3 mL of PBS solution twice and centrifuged again at 250 × g for 3 minutes. Pellets were stored at -80 °C until analyzed by ICP-MS. The assay was performed in triplicate for each treatment group.

For uptake studies after hMATE1 (*SLC47A1*) knockdown, A549 cells were reverse-transfected with Silencer Select siRNA1 or Silencer Select Negative Control #1 scrambled RNA for 48 hours using the RNAiMAX system in Opti-Mem media. Media was replaced with fresh antibiotics-free DMEM/F12K medium and incubation was continued for an additional 24 hours. Cells were then incubated with 100 nM compound **1** at 37 °C for 4 hours, and cell pellets were prepared as described above. The assay was performed in triplicate for each treatment group. Microwave digestions and ICP-MS analysis for Pt were performed as described above.

Assays and sample preparation for cell viability screening

In pyrimethamine competition assays, A549 cells were seeded at a density of 5000 cells per well and allowed to attach for 24 hours. Cells were then pre-treated with 10 or 100 nM pyrimethamine for 20

minutes and subsequently incubated with 100 nM compound **1** or DMF-containing media (control) for 72 hours. No-treatment controls were also included. Assays were run in duplicate with 6 replicates per plate. Cell viability was assessed as described above.

Cell viability in RNAi knockdown assays was assessed by transfecting A549 cells on 96-well plates using a reverse transfection protocol. Briefly, Silencer siRNA, scrambled RNA (Silencer Negative Control #3 siRNA), and lipofectamine (RNAiMAX) were diluted with Opti-Mem prior to mixing in each well to generate a final siRNA concentration of 10 nM. Mixtures were incubated for 20 minutes at room temperature. Cells were then seeded into new wells in DMEM/F12 medium without antibiotics at a density of 5000 cells/well, incubated in the presence of transfection reagent for 24 hours at 37 °C in 5% CO_2, and finally treated with compound **1** at fixed concentrations of 100 nM or 1 μM (or DMF-containing media in control groups) for an additional 24 or 48 hours. Cell viability after 48 and 72 hours was assessed as described above.

In HCT-116 sensitization experiments, cells were seeded at a density of 1100 cells/well in 100 μL of media and allowed to attach overnight. Medium in each well was replaced with fresh medium containing a combination of EED226 and EPZ-6438 to generate final concentrations of 2.5 and 5 μM of each drug. Medium supplemented with epigenetic drugs was replaced every 24 hours and finally removed after 72 hours to begin treatment with compound **1** at concentrations of 1 μM and 10 μM for an additional 72 hours. Cell viability after 72 hours was assessed as described above.

SI References

1 Ikediobi, O. N. *et al.* Mutation analysis of 24 known cancer genes in the NCI-60 cell line set. *Mol. Cancer Ther.* **5**, 2606-2612 (2006).

2 Leroy, B. *et al.* Analysis of TP53 mutation status in human cancer cell lines: a reassessment. *Hum. Mutat.* **35**, 756-765 (2014).

3 McQuin, C. *et al.* CellProfiler 3.0: Next-generation image processing for biology. *PLoS Biol.* **16**, e2005970 (2018).
